# The impact of left ventricular assist devices on kidney function: a systematic review and meta-analysis

**DOI:** 10.1186/s43044-026-00750-7

**Published:** 2026-05-26

**Authors:** Alireza Hosseini, Bahar Darouei, Reza Amani-Beni, Davood Shafie, Sadegh Mazaheri-Tehrani, Mohammad Ali Haghighatpanah, Maryam Heidarpour, Ehsan Amini-Salehi, Seyyed Mohammad Hashemi, Anil Harrison, Irbaz Hameed, Christopher M. Reid, Mohammad Reza Movahed

**Affiliations:** 1 Department of Surgery, School of Medicine, Chamran Hospital, Isfahan University of Medical Sciences, Isfahan, Islamic Republic of Iran; 2 Heart Failure Research Center, Cardiovascular Research Institute, Isfahan University of Medical Sciences, Isfahan, Islamic Republic of Iran; 3 Child Growth and Development Research Center, Research Institute for Primordial Prevention of Non-Communicable Disease, Isfahan University of Medical Sciences, Isfahan, Islamic Republic of Iran; 4 Department of Cardiovascular Surgery, Chamran Heart Center, Isfahan University of Medical Sciences, Isfahan, Islamic Republic of Iran; 5 Isfahan Endocrine and Metabolism Research Center, Isfahan University of Medical Sciences, Isfahan, Islamic Republic of Iran; 6 Guilan University of Medical Sciences, Rasht, Islamic Republic of Iran; 7 Cardiovascular Research Center, Hormozgan University of Medical Sciences, Bandar Abbas, Islamic Republic of Iran; 8 Department of Medicine, Midwestern University, Glendale, AZ USA; 9 Division of Cardiac Surgery, Department of Surgery, Yale University School of Medicine, New Haven, CT USA; 10 Centre of Clinical Research and Education, Curtin University, Perth, WA Australia; 11 Department of Medicine, University of Arizona College of Medicine, Phoenix, USA; 12 Department of Medicine, University of Arizona Sarver Heart Center, Tucson, AZ USA

**Keywords:** Heart failure, Heart-assist devices, Kidney function, Renal health, LVAD

## Abstract

**Background:**

Kidney dysfunction is common in patients with advanced heart failure and is associated with adverse clinical outcomes. Left ventricular assist device (LVAD) implantation may influence renal function through hemodynamic and cardiorenal changes. We performed a meta-analysis to evaluate longitudinal changes in estimated glomerular filtration rate (eGFR) following LVAD implantation.

**Results:**

We systematically searched six databases for studies reporting eGFR before and after LVAD implantation until March 2026. The primary analysis used a multivariate random-effects meta-analysis to jointly pool repeated follow-up estimates while accounting for within-study dependence across time points. Pooled mean changes in eGFR and 95% confidence intervals (CIs) were estimated at 1 week, 2 weeks, 1 month, 3 months, 6 months, 9 months, 1 year, 2 years, and 3 years after implantation. A total of 38 studies contributing 95 effect sizes were included. Compared with baseline, pooled mean eGFR was significantly higher at 1 week (18.83 mL/min/1.73 m²; 95% CI: 13.21, 24.46), 2 weeks (18.29; 95% CI: 12.11, 24.48), 1 month (23.77; 95% CI, 19.48, 28.07), 3 months (15.20; 95% CI: 10.95, 19.46), 6 months (7.97; 95% CI: 3.72, 12.22), 9 months (8.27; 95% CI: 2.10, 14.44), and 1 year (7.34; 95% CI: 2.83, 11.85), but not at 2 or 3 years. In subgroup analyses, studies with baseline eGFR < 60 mL/min/1.73 m² showed significant increases across all evaluated follow-up intervals, whereas those with baseline eGFR ≥ 60 mL/min/1.73 m² showed significant increases only through 3 months.

**Conclusions:**

LVAD implantation was associated with higher eGFR during the early postoperative period, with the largest increase observed at 1 month. However, because most included studies were pre-post analyses without non-LVAD comparator groups, these findings should be interpreted cautiously and do not establish a causal effect. Future research should explore the long-term impact of LVAD on renal health to optimize care strategies and outcomes.

**Supplementary Information:**

The online version contains supplementary material available at 10.1186/s43044-026-00750-7.

## Background

Heart failure (HF) affects about 1–2% of adults in high-income countries and remains a major contributor to global morbidity and mortality [1]. Although many patients can be managed with medical or surgical therapy, some progress to advanced or end-stage HF and require more intensive treatment options [2, 3]. Among these patients, who represent approximately 5–10% of the overall HF population, heart transplantation remains the preferred definitive therapy; However, its use is limited by donor shortage and strict eligibility criteria, leaving many patients either ineligible or unable to receive transplantation in time [2, 4–6].

Accordingly, left ventricular assist devices (LVADs) have become an established therapeutic option as bridge-to-transplantation (BTT) or destination therapy (DT) in patients with advanced HF who are not suitable candidates for transplant [5, 6]. Their broader use has been supported by improved survival and functional capacity in selected patients with end-stage HF, and LVAD implantation has become increasingly common in contemporary practice [7, 8]. Nevertheless, the effects of LVAD support on different organ systems, especially the kidneys, remain incompletely understood.

Kidney dysfunction in advanced HF may be related to pre-existing comorbidities such as hypertension and diabetes, as well as HF-related factors including neurohormonal activation, reduced cardiac output, impaired renal perfusion, and elevated filling pressures [9, 10]. In patients considered for LVAD implantation, renal impairment may range from mild dysfunction to advanced kidney disease [11]. Reduced renal function is also an established adverse prognostic marker in HF; for example, each 1 mL/min decrease in creatinine clearance below 60 ml/min has been associated with a higher risk of mortality [12]. The impact of LVAD on the estimated glomerular filtration rate (eGFR), a key indicator of kidney function, is complex, with studies reporting varying results [10, 13, 14]. While some reports describe an early rise in eGFR after implantation, others suggest that this improvement may attenuate during longer-term follow-up [15].

Although this topic has been increasingly studied, a quantitative synthesis of longitudinal changes in GFR after LVAD implantation remains limited. Therefore, we performed this meta-analysis to evaluate short- and long-term changes in kidney function after LVAD implantation, with a particular focus on overall eGFR trajectory over time.

## Materials and methods

### Protocol and registration

This study followed the Preferred Reporting Items for Systematic Reviews and Meta-analysis (PRISMA) guidelines [16] and was registered in the PROSPERO database (registration number CRD42024497250).

### Search strategy

We systematically searched major databases from inception until December 3, 2023 and updated the search on March 2, 2026, including MEDLINE, Embase, Scopus, Web of Science, Cochrane Central Register of Controlled Trials, and ClinicalTrials.gov. We manually searched Google and Google Scholar and screened reference lists of relevant reviews. The systematic search utilized the search query provided in **Table S1** to identify pertinent literature. Although the search encompassed all languages, the final analysis was limited to articles published in English. No filters were applied to limit study design initially, and studies were screened based on relevance and eligibility criteria. We excluded reviews, case reports, commentaries, editorials, conference papers, abstracts, letters to the editor, errata, and studies on animal or cellular models.

### Eligibility criteria

Three reviewers (B.D., S.M.T., and M.A.H.) independently screened the literature using predefined eligibility criteria. Studies were eligible if they met the following criteria: (1) prospective or retrospective cohort studies evaluating changes in eGFR before and after LVAD implantation, reported as mean ± standard deviation (SD); (2) the intervention consisted solely of durable LVAD implantation; (3) the study population included adult patients (≥ 18 years); and (4) the duration of follow-up after LVAD implantation was reported.

Studies were excluded if they included patients younger than 18 years or pregnant women; if LVAD implantation was combined with other surgical procedures; if the study involved other mechanical circulatory support devices (e.g., percutaneous ventricular assist devices, right ventricular assist devices, or biventricular assist devices); or if overlapping patient populations were identified. In cases of potential cohort overlap, the study with the most comprehensive dataset or largest sample size was retained.

### Study selection

Three reviewers (B. D., E.A.S., and S.M.H.) independently screened titles and abstracts for duplication and selected relevant articles. Full-text articles were further reviewed to confirm eligibility. We employed Endnote software version 21 to validate the screened records, and in instances where necessary data were lacking, the authors were contacted via email for further information.

### Data extraction

From the eligible articles, the following data were retrieved: study characteristics (first author, year of publication, study period, study design, and country), sample size, participant demographics (percentage of females, and age) LVAD device type, LVAD implantation indication, HF etiology, baseline left ventricular ejection fraction (LVEF), baseline creatinine, percentage of renal replacement therapy, eGFR method, and outcome (duration of follow-up, and mean ± c eGFR before and after LVAD implantation). Any conflicts encountered during screening or data extraction were managed by discussion with a third reviewer (R.A.B.). Microsoft Excel and Word were used for data extraction and management.

### Quality assessment

Three independent reviewers (A.H., D.Sh., and M.H.) appraised the methodological quality of the included studies was assessed using the National Institutes of Health (NIH Quality) Quality Assessment Tool for Before-After (Pre-Post) Studies with No Control Group, which is specifically designed for single-arm pre-post studies [17]. The tool evaluates 12 domains, including clarity of the study objective, eligibility criteria, representativeness of participants, sample size justification, consistency of intervention delivery, validity and reliability of outcome measures, blinding of outcome assessors, adequacy of follow-up, repeated outcome assessment before and after the intervention, and appropriateness of the statistical analysis. Each item was judged as Yes, No, Cannot Determine/Not Reported/Not Applicable, according to the NIH guidance. For overall study quality, we calculated the proportion of “Yes” responses among applicable items only, excluding items rated as not applicable from the denominator. Studies were then categorized as poor (< 50%), fair (50%−75%), or good (≥ 75%). Any disagreements were resolved by consulting a fourth reviewer (R.A.B.).

### Statistical analysis

The primary outcome was the mean change in eGFR from baseline to each reported follow-up time point after LVAD implantation. For each study and follow-up time point, the effect size was calculated as the difference between the follow-up mean eGFR and the baseline mean eGFR. When studies reported baseline and follow-up means and SD but did not report the SD of the change score, the SD of the change was imputed using the conventional formula:

$$\:S{D}_{change}=\sqrt{S{D}_{baseline}^{2}+S{D}_{follow\mathrm{-}up}^{2}-2r\left(S{D}_{baseline}\right)\left(S{D}_{follow\mathrm{-}up}\right)}$$

assuming a common within-study baseline-to-follow-up correlation of *r* = 0.5 [18, 19]. The standard error of the mean change was then derived from the corresponding standard deviation and sample size.

Our primary analysis used a multivariate random-effects meta-analysis to jointly pool repeated follow-up estimates from the same study while accounting for within-study dependence across time points. The model was fitted using restricted maximum likelihood (REML) with an exchangeable random-effects covariance structure. Studies were allowed to contribute data to any available follow-up time point, and studies with incomplete follow-up schedules were retained in the multivariate model under the assumption that outcomes were missing at random conditional on the observed data. Pooled mean changes and 95% confidence intervals (CIs) were estimated for 1 week, 2 weeks, 1 month, 3 months, 6 months, 9 months, 1 year, 2 years, and 3 years. When studies reported follow-up at nonstandard time points, these were harmonized to the nearest prespecified follow-up category based on the reported mean or median follow-up time. Follow-up times reported in days were converted to months using 30 days per month before assignment. Heterogeneity in the multivariate model was assessed using the Jackson-White-Riley (JWR) I² and R statistics, both overall and separately for each follow-up time point. The omnibus heterogeneity statistic from the multivariate model was also reported. Because the multivariate analysis was intended to model the overall trajectory while accounting for repeated measures within studies, it served as the main analysis for inference.

Exploratory subgroup analyses were conducted using multivariate random-effects models stratified by baseline kidney function, categorized according to study-level mean baseline eGFR as < 60 versus ≥ 60 mL/min/1.73 m², and eGFR estimation method, including Modification of Diet in Renal Disease (MDRD), Chronic Kidney Disease Epidemiology Collaboration (CKD-EPI), and creatinine clearance. Exploratory multivariate meta-regression analyses were also performed to examine whether study-level mean age and female proportion were associated with changes in eGFR over time. To improve interpretability, mean age was scaled per 10-year increase and female proportion was scaled per 10%-point increase. Meta-regression coefficients were estimated separately for each follow-up time point within the multivariate framework.

A risk-of-bias sensitivity analysis was performed by restricting the multivariate meta-analysis to studies rated as fair or good quality according to the NIH Quality Assessment Tool for Before-After Studies with No Control Group, excluding studies rated as poor quality. To evaluate model adequacy and robustness, we also examined observed-versus-fitted plots and Q-Q plots of standardized predicted random effects. Studies appearing potentially outlying on diagnostic assessment were further evaluated by one-by-one sensitivity analyses, in which the multivariate model was re-estimated after excluding each study individually to determine whether any single study materially changed the pooled estimates or overall interpretation.

As secondary analyses, separate follow-up-specific random-effects meta-analyses were performed using the REML method. When fewer than 10 studies were available for a given follow-up time point, inference was based on the Knapp-Hartung adjustment. For these one-by-one analyses, we generated forest plots and calculated standard heterogeneity statistics, including I² and τ². Potential small-study effects (publication bias) were explored in the follow-up-specific secondary analyses. Funnel plots were generated for follow-up times with more than 10 studies, and Egger’s regression test, Begg’s rank correlation test, and trim-and-fill analysis were performed for each time point. All statistical tests were two-sided, and *p* < 0.05 was considered statistically significant. All statistical analyses were performed using Stata version 18 (StataCorp LLC, College Station, TX, USA).

## Results

### Study selection

**Fig. 1:**
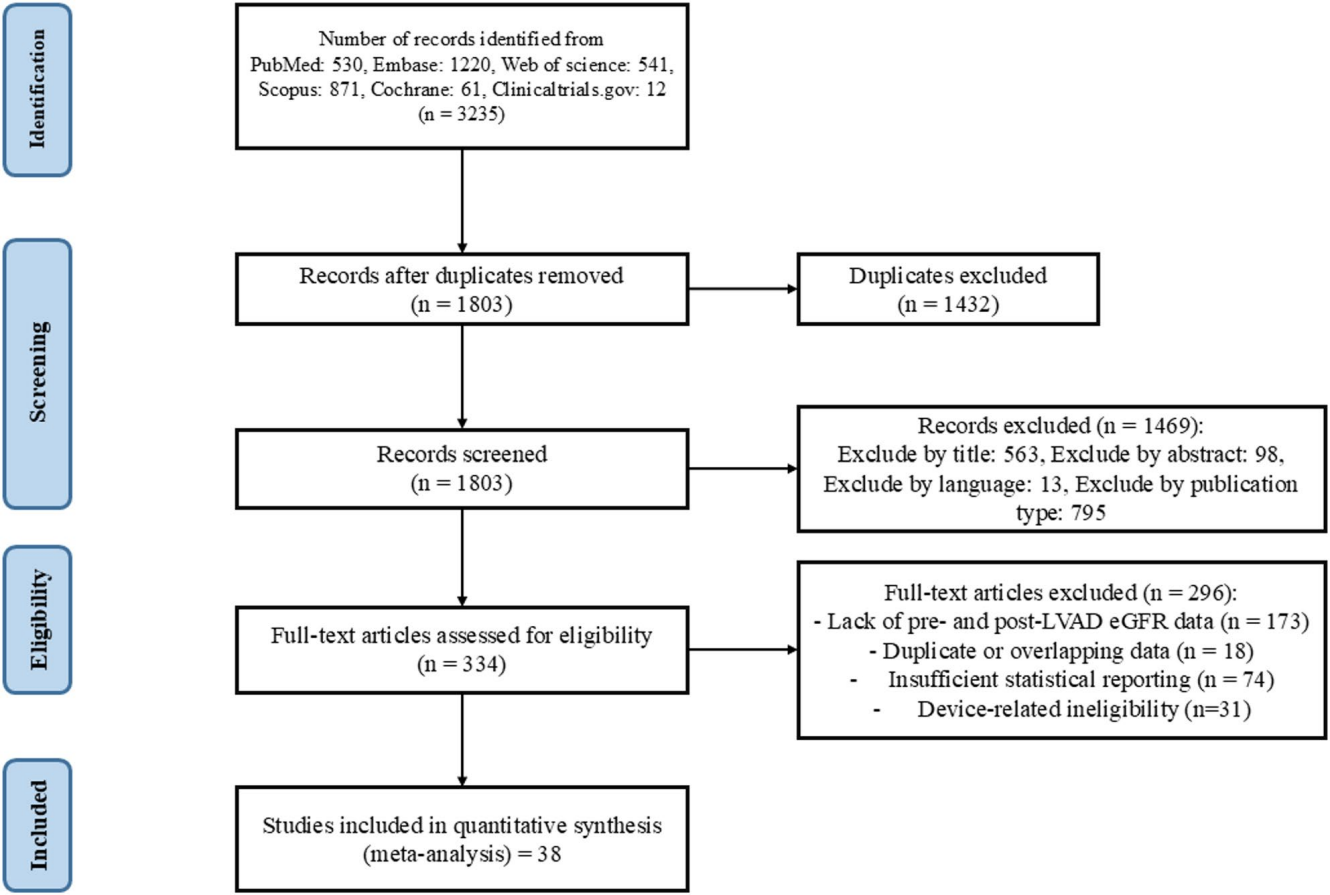
PRISMA flowchart of the literature search and selection of studies

A total of 3235 studies were initially included; after duplicate removal, 1803 articles remained. Title and abstract screening reduced the pool to 334 articles, which underwent full-text review. Full-text articles were primarily excluded due to overlapping cohorts (*n* = 18), lack of baseline or follow-up eGFR data (*n* = 173), insufficient statistical reporting (*n* = 74) or device-related ineligibility (*n* = 31). Ultimately, 38 studies met the inclusion criteria for meta-analysis (Fig. 1) [9, 10, 13–15, 20–52]. Of these, 27 were retrospective cohort studies, and 11 were prospective cohort studies.

### Characteristics of the included studies

Table S2 provides a summary of the general characteristics of the studies included. This meta-analysis included 16,058 patients with HF from 38 cohort studies. Among studies reporting HF etiology, ischemic cardiomyopathy accounted for 46.8% of cases, whereas non-ischemic etiologies accounted for 53.2%. LVEF was reported in 20 studies and was markedly reduced overall, with study-level mean values clustering around 18%. Post-LVAD renal replacement therapy was reported in 16 studies and was observed in approximately 14.5% of patients.

LVAD indication was reported in 26 studies, and BTT was the most frequently reported indication. However, some studies did not clearly differentiate DT and BTD, limiting more detailed subgroup description. Renal function was most commonly assessed using the MDRD Eq. (16 studies), followed by the CKD-EPI Eq. (8 studies), creatinine clearance (5 studies), and the Cockcroft-Gault formula (1 study). The quality assessment is shown in Table S3. Overall, 10 cohort studies were rated as good quality, 25 studies were rated as fair quality, and 3 studies were classified as poor quality.

### Mean change in eGFR

**Fig. 2:**
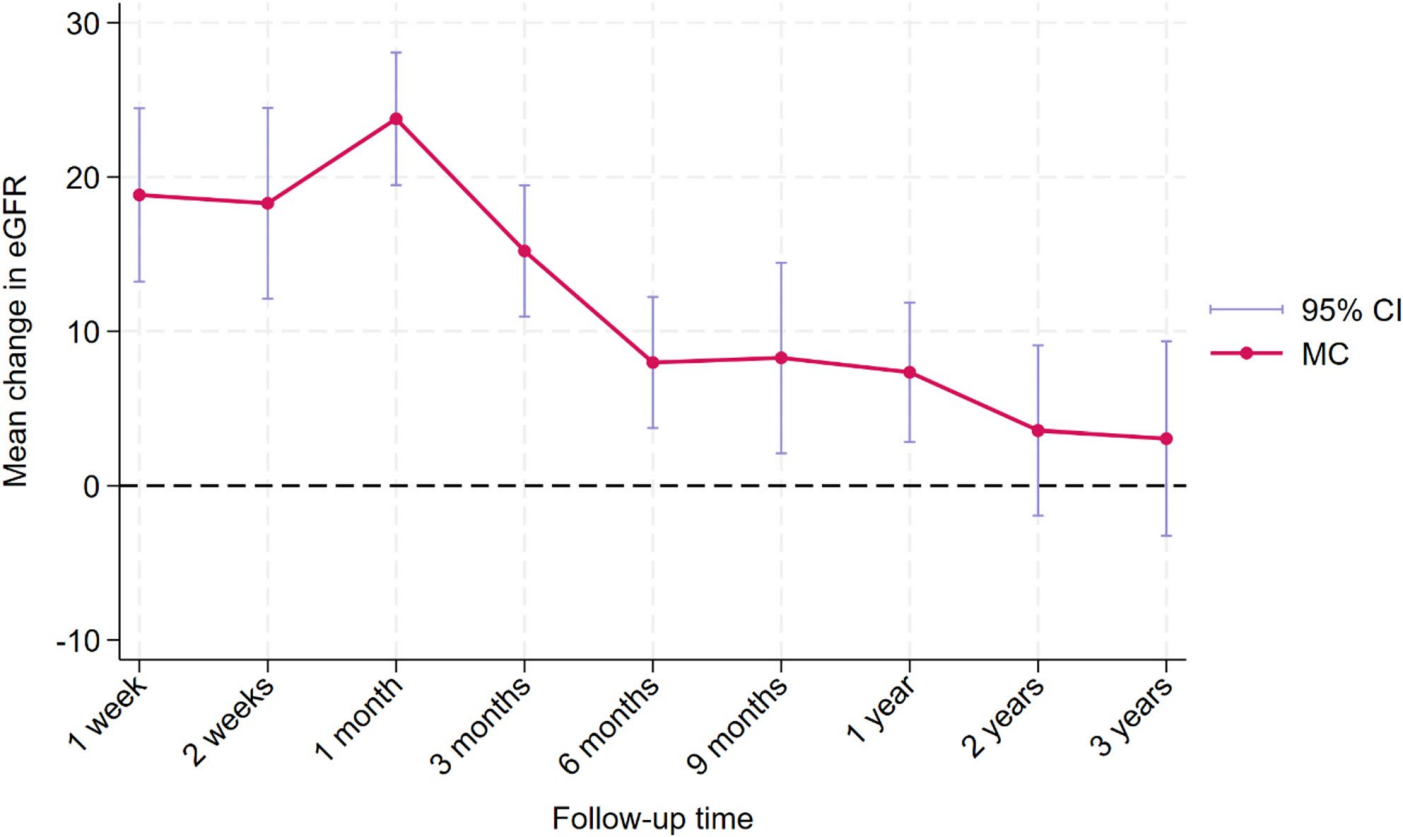
Trajectory of mean change of eGFR from baseline during the follow-up time. Note: All effect sizes and confidence intervals displayed on the vertical axis represent the change in eGFR, measured in mL/min/1.73 m²

In the primary analysis, a multivariate random-effects meta-analysis including 95 effect sizes from 38 studies showed that the pooled mean change in eGFR was significantly positive during the early and intermediate follow-up periods (Fig. 2 and Table 1). The pooled mean change was 18.83 (95% CI: 13.21, 24.46) at 1 week, 18.29 (95% CI: 12.11, 24.48) at 2 weeks, and reached its highest value at 1 month with a pooled mean change of 23.77 (95% CI: 19.48, 28.07). Thereafter, the pooled effect gradually attenuated but remained statistically significant at 3 months (β = 15.20: 95% CI 10.95, 19.46), 6 months (β = 7.97; 95% CI: 3.72, 12.22), 9 months (β = 8.27; 95% CI: 2.10, 14.44), and 1 year (β = 7.34; 95% CI: 2.83, 11.85). In contrast, the pooled mean changes at 2 years (β = 3.57; 95% CI: −1.94, 9.08) and 3 years (β = 3.05; 95% CI: −3.25, 9.35) were no longer statistically significant. Substantial heterogeneity was observed across all follow-up times. Using the Jackson-White-Riley method, I² ranged from 77.34% at 2 weeks to 98.42% at 1 month, while the joint multivariate heterogeneity was also high (I² = 89.58%, *R* = 3.10).

**Table 1:** Primary multivariate random-effects meta-analysis of mean change in eGFR across follow-up

Follow-up time	No. of studies	Pooled mean change in eGFR β (95% CI)	*p*-value	JWR I² (%)	*R*
1 week	7	18.83 (13.21 to 24.46)	< 0.001	96.17	5.11
2 weeks	5	18.29 (12.11 to 24.48)	< 0.001	77.34	2.10
1 month	19	23.77 (19.48 to 28.07)	< 0.001	98.42	7.95
3 months	18	15.20 (10.95 to 19.46)	< 0.001	94.96	4.45
6 months	19	7.97 (3.72 to 12.22)	< 0.001	94.45	4.25
9 months	5	8.27 (2.10 to 14.44)	0.009	89.76	3.12
1 year	13	7.34 (2.83 to 11.85)	0.001	94.12	4.12
2 years	5	3.57 (−1.94 to 9.08)	0.204	91.69	3.47
3 years	4	3.05 (−3.25 to 9.35)	0.342	88.67	2.97

eGFR, estimated glomerular filtration rate; JWR I², Jackson-White-Riley I²; No., number

### Subgroup analysis

**Fig. 3:**
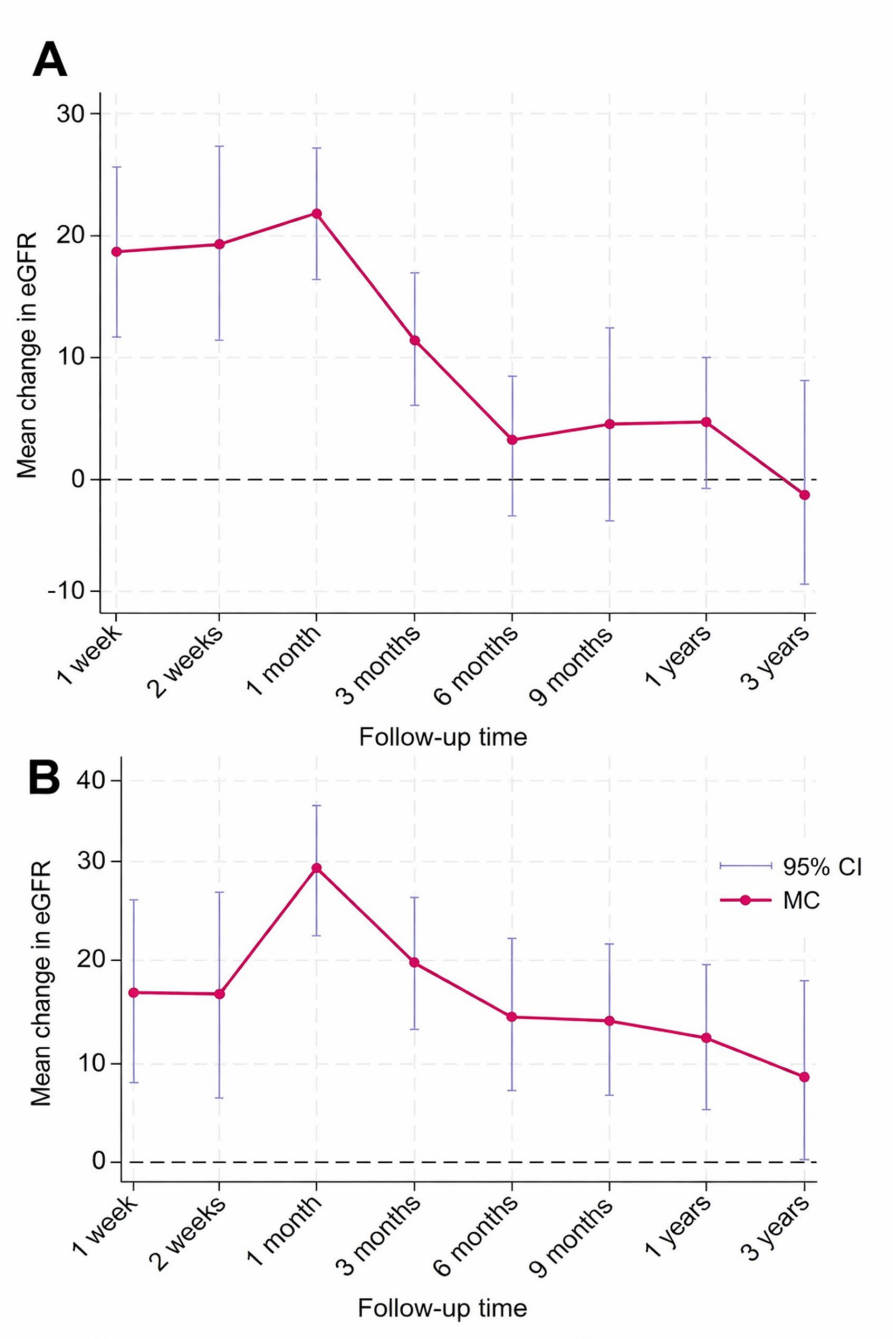
Trajectory of mean change of eGFR from baseline during the follow-up time in studies with (A) baseline mean eGFR ≥ 60 mL/min/1.73m^2^ and (B) baseline mean eGFR < 60 mL/min/1.73m^2^. Note: All effect sizes and confidence intervals displayed on the vertical axis represent the change in eGFR, measured in mL/min/1.73 m²

In subgroup analysis according to baseline kidney function, studies with baseline eGFR < 60 mL/min/1.73 m² showed significantly positive pooled mean changes in eGFR at all evaluated follow-up times, from 1 week through 3 years. The largest pooled increase was observed at 1 month (β = 27.15; 95% CI 20.83, 33.48). In contrast, in studies with baseline eGFR ≥ 60 mL/min/1.73 m², pooled mean change in eGFR was significantly positive from 1 week through 3 months, but was no longer statistically significant from 6 months onward (Fig. 3 and Table 2).

**Table 2:** Multivariate subgroup analysis according to baseline eGFR

Follow-up time	Baseline eGFR < 60 β (95% CI)	*p*-value	Baseline eGFR ≥ 60 β (95% CI)	*p*-value
1 week	16.59 (7.96 to 25.22)	< 0.001	18.64 (11.47 to 25.81)	< 0.001
2 weeks	16.27 (7.30 to 25.25)	< 0.001	19.23 (11.20 to 27.26)	< 0.001
1 month	27.15 (20.83 to 33.48)	< 0.001	21.82 (16.54 to 27.11)	< 0.001
3 months	19.30 (13.30 to 25.30)	< 0.001	11.24 (5.64 to 16.83)	< 0.001
6 months	13.87 (7.81 to 19.93)	< 0.001	2.06 (−3.42 to 7.53)	0.462
9 months	14.87 (7.29 to 22.45)	< 0.001	3.65 (−5.28 to 12.57)	0.423
1 year	11.55 (5.33 to 17.78)	< 0.001	4.72 (−1.16 to 10.60)	0.116
2 years	10.46 (3.64 to 17.29)	0.003	−1.86 (−11.01 to 7.29)	0.691
3 years	8.12 (0.25 to 15.99)	0.043	0.44 (−9.62 to 10.50)	0.932

eGFR, estimated glomerular filtration rate; CI, confidence interval

In subgroup analysis according to eGFR estimation method, studies using MDRD showed significantly positive pooled mean changes in eGFR from 1 week through 1 year, whereas the estimates at 2 years and 3 years were no longer statistically significant. Studies using CKD-EPI showed significant increases at 1 week, 1 month, and 3 months, but not at the remaining follow-up times. In studies using creatinine clearance, pooled mean change in eGFR was significantly positive from 2 weeks through 1 year, with the largest estimate observed at 1 year (β = 31.00; 95% CI: 19.77, 42.23). No pooled estimates were available for creatinine clearance at 2 or 3 years because of insufficient data (Table 3).

**Table 3:** Multivariate subgroup analysis according to eGFR estimation method

Follow-up time	MDRD, β (95% CI)	*p*-value	CKD-EPI, β (95% CI)	*p*-value	Creatinine clearance, β (95% CI)	*p*-value
1 week	23.02 (15.22 to 30.82)	< 0.001	20.06 (5.44 to 34.67)	0.007	7.88 (−2.10 to 17.86)	0.122
2 weeks	20.39 (11.71 to 29.07)	< 0.001	12.10 (−2.44 to 26.63)	0.103	11.44 (1.01 to 21.88)	0.032
1 month	28.36 (22.41 to 34.31)	< 0.001	15.06 (4.44 to 25.68)	0.005	21.87 (12.48 to 31.25)	< 0.001
3 months	18.00 (11.96 to 24.04)	< 0.001	11.72 (2.14 to 21.30)	0.016	21.18 (11.18 to 31.19)	< 0.001
6 months	12.04 (6.07 to 18.01)	< 0.001	1.47 (−9.00 to 11.93)	0.784	18.68 (7.11 to 30.25)	0.002
9 months	12.32 (2.67 to 21.96)	0.012	12.37 (−10.69 to 35.43)	0.293	20.47 (8.95 to 31.99)	< 0.001
1 year	8.18 (1.57 to 14.79)	0.015	1.23 (−8.36 to 10.81)	0.802	31.00 (19.77 to 42.23)	< 0.001
2 years	6.81 (−0.15 to 13.77)	0.055	−3.83 (−16.75 to 9.09)	0.561	–	–
3 years	5.80 (−2.33 to 13.94)	0.162	−2.58 (−15.70 to 10.54)	0.700	–	–

eGFR, estimated glomerular filtration rate; CI, confidence interval; MDRD, Modification of Diet in Renal Disease Study Equation; CKD-EPI, Chronic Kidney Disease Epidemiology Collaboration Equation

### Sensitivity analysis

In sensitivity analysis restricted to studies with fair or good NIH risk-of-bias ratings, the overall pattern of results remained unchanged. Positive and statistically significant pooled mean increases in eGFR were observed from 1 week through 1 year, with the largest estimate at 1 month (β = 23.97; 95% CI: 19.41, 28.53). The pooled estimates at 2 years and 3 years were not statistically significant. These findings were consistent with the primary multivariate analysis (Table S4).

### Meta-regression analysis

Exploratory multivariate meta-regression showed no statistically significant overall association for either age or female proportion. A per 10-year increase in mean age was associated with a smaller mean change in eGFR at 2 weeks (β = −10.82; 95% CI: −19.93, −1.72; *p* = 0.020), while the association at 1 month was borderline (β = −7.58, 95% CI −15.37 to 0.21; *p* = 0.057). A per 10% increase in female proportion was associated with a greater mean change in eGFR at 3 months (β = 8.83; 95% CI: 0.91, 16.75; *p* = 0.029), whereas no other time-specific associations were statistically significant (Table S5).

### Model diagnostics

Observed-versus-fitted plots showed no major model misfit (Figure S1). Q-Q plots suggested a small number of potentially outlying studies at selected follow-up times; however, one-by-one sensitivity analyses excluding these studies did not materially change the pooled estimates or overall interpretation (Figure S2).

### Secondary time-point-specific analyses

As a secondary analysis to assess the robustness of the multivariate findings, we performed separate random-effects meta-analyses for each follow-up time point. These analyses yielded results that were broadly consistent with the primary multivariate model, with the largest pooled increases in eGFR observed during the early follow-up period and attenuation of the effect over time. Forest plots for each time point are provided in the Supplementary Material (Figure S3-S11).

### Small-study effects

Small-study effects were also explored in the time-point-specific analyses. Funnel plots were constructed for follow-up times with more than 10 contributing studies, while Egger’s test, Begg’s test, and trim-and-fill analyses were performed for all follow-up times. These analyses did not suggest a pattern that materially changed the overall interpretation, although they were considered exploratory because of the limited number of studies at several follow-up points (Figure S12-S15).

## Discussion

In this meta-analysis, LVAD implantation was associated with an early increase in eGFR, with the largest improvement observed at 1 month, followed by attenuation over time; the association remained significant through 1 year but not at 2 and 3 years. Subgroup analyses suggested that this pattern differed by baseline kidney function, with more sustained improvement in studies including patients with lower baseline eGFR, while studies with higher baseline eGFR showed only early improvement. Results were also examined according to eGFR estimation method and were generally directionally consistent, although the timing and persistence of significance varied across formulas. Separate time-point-specific meta-analyses supported the primary multivariate results. Exploratory meta-regression identified limited time-specific associations for age and female proportion. Overall, these results support a heterogeneous and time-dependent pattern of renal function change after LVAD implantation and should be interpreted in light of the observational design of the included studies.

In post-LVAD patients, variations in renal function are common, with some individuals achieving stable or better eGFR levels, an essential parameter for renal filtration and prognostication in this population [53, 54]. For various reasons, physicians need to understand the alterations in renal function that occur after LVAD implantation. Understanding these changes is crucial for several clinical decisions, such as the need for chronic renal replacement therapy, combined heart-kidney transplantation, and heart transplant candidacy [15, 55]. In the absence of definitive tests for predicting renal dysfunction reversibility in HF patients, measuring the expected recovery of renal function after LVAD implantation is critical.

Our meta-analysis showed the largest pooled increase in eGFR at 1 month after LVAD implantation [15]. This early pattern is consistent with prior LVAD cohorts reporting short-term improvement in creatinine-based kidney function after implantation, particularly among patients with impaired baseline renal function. However, because the included studies did not directly measure renal perfusion, neurohormonal activation, or venous congestion, the mechanisms underlying the early postoperative change in eGFR is yet to be determined. Additionally, a reduction in muscle mass shortly after LVAD implantation may transiently lower creatinine levels, leading to an overestimation of eGFR at one month [9].

Beyond the early postoperative peak, published longitudinal studies suggest that kidney function after LVAD implantation often attenuates toward baseline rather than showing uniform durable improvement. Yoshioka et al. reported that the initial renal improvement during prolonged continuous-flow LVAD support was largely transient and returned toward baseline over longer follow-up [39]. Bujo et al. similarly found improvement at 1 month with return to preoperative levels by 6 months and near-baseline values thereafter [56]. In addition, Walther et al. demonstrated that kidney function after LVAD implantation may follow several distinct trajectories rather than a single durable pattern and important inter-patient heterogeneity [57]. Consistent with this literature, our updated overall analysis showed that the pooled increase in eGFR remained statistically significant through 1 year but was no longer statistically significant at 2 or 3 years. Moreover, because the later estimates were based on limited numbers of studies, they should be interpreted cautiously and should not be taken as evidence of durable long-term renal benefit. Potential contributors to later kidney-function decline, such as right-sided dysfunction or persistent venous congestion, have been described in individual LVAD cohorts [58], but these factors were not measured consistently across the studies included in our meta-analysis. Accordingly, they should be regarded as possible explanations rather than causal inferences from the present study.

The attenuation of kidney function beyond 1 year may also reflect post-implant complications and treatment-related exposures reported in the LVAD literature. In particular, driveline infections are common during longer-term LVAD support and may require prolonged courses of broad-spectrum antibiotics, some of which can be nephrotoxic, especially in patients with underlying renal vulnerability [59–62]. However, recurrent infection, antibiotic exposure, and treatment-related nephrotoxicity were not captured systematically across the included studies. In addition, because creatinine-based eGFR may be influenced by changes in muscle mass after LVAD implantation, part of the early apparent improvement may diminish over time as muscle mass recovers during rehabilitation.

When interpreting longitudinal renal function post-LVAD, it is critical to recognize the inherent limitations of relying solely on serum creatinine-based eGFR. In the LVAD population, serum creatinine kinetics are heavily confounded by significant postoperative fluid shifts and, most notably, profound muscle wasting. As demonstrated by Pinsino et al., the apparent early improvement in creatinine-based eGFR following LVAD implantation may be largely driven by GFR-independent reductions in muscle mass rather than true intrinsic renal recovery [33]. Consequently, alternative biomarkers that are less dependent on muscle mass, such as Cystatin C, have been shown to provide a more reliable and accurate estimation of true renal function and are superior predictors of early postoperative adverse outcomes in LVAD recipients [33].

In our subgroup analysis, studies with baseline eGFR < 60 mL/min/1.73 m² showed significant increases in eGFR across all follow-up intervals through 3 years, whereas studies with baseline eGFR ≥ 60 mL/min/1.73 m² showed significant increases only through 3 months. The greater increase in eGFR observed in studies with mean baseline eGFR < 60 mL/min/1.73 m² may reflect a higher burden of potentially reversible, hemodynamically mediated cardiorenal dysfunction [15, 30, 31].

In advanced heart failure, renal dysfunction is often associated with reduced cardiac output and elevated venous pressures, both of which may contribute to cardiorenal dysfunction [15, 63]. Studies enrolling populations with lower mean preoperative eGFR may represent cohorts with a greater burden of potentially reversible cardiorenal dysfunction. Accordingly, the larger postoperative increase in eGFR observed in studies with baseline eGFR < 60 may reflect a greater capacity for renal recovery after LVAD implantation [63]. However, although this pattern may indicate greater potential for sustained renal improvement in studies with lower mean baseline kidney function, the analysis was based on study-level mean eGFR and is therefore vulnerable to ecological bias. Moreover, substantial between-study heterogeneity, differences in patient and device characteristics, variation in perioperative care, and the absence of non-LVAD comparator groups limit causal inference.

Similar to all meta-analyses, there exist certain inherent limitations: First, the analysis was conducted on retrospective or prospective studies that were susceptible to selection bias. Moreover, there was potential heterogeneity in some variables such as baseline age, sex, baseline eGFR, methods for eGFR calculation, underlying chronic kidney disease and etiology of HF. Differences in LVAD device types (CF vs. pulsatile-flow) may have influenced renal outcomes. While continuous-flow LVADs have better durability, they lack pulsatility, which may impact renal perfusion and contribute to long-term renal function decline in some patients. Renal function in the included cohorts was almost exclusively assessed using creatinine-based equations (e.g., MDRD, CKD-EPI). Because alternative and potentially more accurate renal biomarkers, such as Cystatin C were not routinely assessed or reported in the primary studies, our analysis could not adjust for the confounding effects of postoperative muscle wasting and fluid shifts on eGFR trajectories Finally, the number of studies contributing to several follow-up time points was modest, which may have reduced the precision of heterogeneity estimates and limited the power of statistical tests for small-study effects. Accordingly, funnel plot asymmetry, Egger’s test, Begg’s test, and trim-and-fill analyses should be interpreted cautiously, particularly for time points with fewer contributing studies.

## Conclusions

In this meta-analysis of 38 observational cohort studies, LVAD implantation was associated with a significant increase in eGFR during the early and intermediate follow-up periods, with the greatest pooled increase observed at 1 month after implantation. In the overall analysis, this association attenuated over time and was no longer statistically significant at 2 or 3 years. Subgroup analyses suggested that studies with lower mean baseline eGFR (< 60 mL/min/1.73 m²) showed more sustained increases in eGFR across follow-up, whereas studies with higher mean baseline eGFR (≥ 60 mL/min/1.73 m²) showed significant increases mainly during the early post-implantation period. These findings should be interpreted cautiously given the observational pre-post nature of most included studies, the absence of non-LVAD comparator groups, and the substantial between-study heterogeneity; moreover, regression to the mean, perioperative hemodynamic optimization, and survival bias may have contributed to the observed pattern. Comparative studies with longer follow-up are needed to better define the long-term renal trajectory after LVAD implantation.

## Supplementary Material

Supplementary Material 1. The Supplementary Material includes: Table S1, search strategy; Table S2, characteristics of the studies included in the meta-analysis; Table S3, risk-of-bias assessment using the NIH Quality Assessment Tool for Before-After (Pre-Post) Studies With No Control Group; Table S4, sensitivity analysis restricted to studies with fair or good risk of bias; Table S5, multivariate meta-regression of mean age and female proportion in relation to change in eGFR across follow-up; Figure S1, observed-versus-fitted plots for the multivariate random-effects meta-analysis; Figure S2, Q-Q plots of standardized predicted random effects; Figures S3–S11, forest plots of pooled mean change in eGFR across follow-up time points; and Figures S12–S15, funnel plots for assessment of small-study effects.

## Data Availability

No datasets were generated or analysed during the current study.

## Abbreviations

LVAD: Left ventricular assist device
eGFR: Estimated glomerular filtration rate
CI: Confidence interval
HF: Heart failure
BTT: Bridge-to-transplant
DT: Destination therapy
SD: Standard deviation
LVEF: Left ventricular ejection fraction
AKI: Acute kidney injury
MDRD: Modification of diet in renal disease
