# Supplementary material for: The impact of left ventricular assist devices on kidney function: a systematic review and meta-analysis
Source: Egypt Heart J. 2026 May 26;78:39. doi: 10.1186/s43044-026-00750-7 (PMC13212856; doi:10.1186/s43044-026-00750-7)
Supplement: Supplementary file 1 — Supplementary Material 1. The Supplementary Material includes: Table S1, search strategy; Table S2, characteristics of the studies included in the meta-analysis; Table S3, risk-of-bias assessment using the NIH Quality Assessment Tool for Before-After (Pre-Post) Studies With No Control Group; Table S4, sensitivity analysis restricted to studies with fair or good risk of bias; Table S5, multivariate meta-regression of mean age and female proportion in relation to change in eGFR across follow-up; Figure S1, observed-versus-fitted plots for the multivariate random-effects meta-analysis; Figure S2, Q-Q plots of standardized predicted random effects; Figures S3–S11, forest plots of pooled mean change in eGFR across follow-up time points; and Figures S12–S15, funnel plots for assessment of small-study effects. [file 43044_2026_750_MOESM1_ESM.docx]

**Supplementary Material**

**Table of Contents**

[Table S1. Search strategy 2](#_Toc224507371)

[Table S2. Characteristics of the studies included in meta-analysis. 4](#_Toc224507372)

[Table S3. Risk of bias assessment using the NIH Quality Assessment Tool for Before-After (Pre-Post) Studies with No Control Group. 10](#_Toc224507373)

[Table S4. Sensitivity analysis restricted to studies with fair or good risk of bias. 12](#_Toc224507374)

[Table S5. Multivariate meta-regression of mean age and female proportion in relation to change in eGFR across follow-up. 13](#_Toc224507375)

[Figure S1. Observed-versus-fitted plots for the multivariate random-effects meta-analysis. 14](#_Toc224507376)

[Figure S2. Q-Q plots of standardized predicted random effects from the multivariate random-effects meta-analysis. 15](#_Toc224507377)

[Figure S3. Forest plot of the random-effects meta-analysis showing the pooled mean change in eGFR at 1 week. 16](#_Toc224507378)

[Figure S4. Forest plot of the random-effects meta-analysis showing the pooled mean change in eGFR at 2 weeks. 17](#_Toc224507379)

[Figure S5. Forest plot of the random-effects meta-analysis showing the pooled mean change in eGFR at 1 month. 18](#_Toc224507380)

[Figure S6. Forest plot of the random-effects meta-analysis showing the pooled mean change in eGFR at 3 months. 19](#_Toc224507381)

[Figure S7. Forest plot of the random-effects meta-analysis showing the pooled mean change in eGFR at 6 months 20](#_Toc224507382)

[Figure S8. Forest plot of the random-effects meta-analysis showing the pooled mean change in eGFR at 9 months. 21](#_Toc224507383)

[Figure S9. Forest plot of the random-effects meta-analysis showing the pooled mean change in eGFR at 1 year. 22](#_Toc224507384)

[Figure S10. Forest plot of the random-effects meta-analysis showing the pooled mean change in eGFR at 2 years. 23](#_Toc224507385)

[Figure S11. Forest plot of the random-effects meta-analysis showing the pooled mean change in eGFR at 3 years. 24](#_Toc224507386)

[Figure S12. Funnel plot for assessment of small-study effects in the random-effects meta-analysis of mean change in eGFR at 1 month. 25](#_Toc224507387)

[Figure S13. Funnel plot for assessment of small-study effects in the random-effects meta-analysis of mean change in eGFR at 3 months. 26](#_Toc224507388)

[Figure S14. Funnel plot for assessment of small-study effects in the random-effects meta-analysis of mean change in eGFR at 6 months. 27](#_Toc224507389)

[Figure S15. Funnel plot for assessment of small-study effects in the random-effects meta-analysis of mean change in eGFR at 1 year. 28](#_Toc224507390)

# Table S1. Search strategy

| **Databases** | **Field** | **Search query** |
| --- | --- | --- |
| Medline | All fields | ("ventricular assist device*" OR "cardiac assist device*" OR "Heart-Assist Devices"[Mesh] OR "CF-LVAD" OR "continuous-flow pump*" OR "pulsatile pump*" OR LVAD OR "left ventricular assist device*") AND ("Glomerular Filtration Rate"[MESH] OR "Kidney Function Tests"[MESH] OR "Renal Elimination"[MESH] OR "glomerular filtration" OR "GFR" OR "eGFR" OR "kidney function" OR "renal function" OR "kidney elimination" OR "creatinine clearance" OR "CCR" OR "CrCl" OR "eCCR" OR "inulin clearance" OR "cystatin C clearance" OR "urea clearance" OR "glomerulus filtration rate") |
| EMBASE | Title / abstract / keyword | ('heart-assist device*' OR 'heart assist device*' OR 'left ventricular assist device*' OR 'continuous-flow pump*' OR 'pulsatile pump*' OR 'heart assisting device*' OR 'LVAD*' OR 'CF-LVAD' OR 'cardiac assist device*' OR 'ventricular assist device*' OR 'ventricular-assist device*') AND (‘Glomerular Filtration Rate’ OR 'glomerulus filtration rate' OR 'kidney function' OR ‘renal function’ OR 'creatinine clearance' OR 'eGFR' OR 'GFR' OR 'renal elimination' OR 'kidney elimination' OR 'CCR' OR 'CrCl' OR 'eCCR' OR 'inulin clearance' OR 'cystatin C clearance' OR 'urea clearance') |
| Web of Science | All fields | ("ventricular assist device*" OR "cardiac assist device*" OR "Heart-Assist Devices" OR "CF-LVAD" OR "continuous-flow pump*" OR "pulsatile pump*" OR "heart assisting device*" OR "LVAD*" OR "CF-LVAD'" OR "cardiac assist device*" OR "ventricular assist device*" OR "ventricular-assist device*") AND ("Glomerular Filtration Rate" OR "Kidney Function Test" OR "glomerular filtration" OR "GFR" OR "eGFR" OR "kidney function" OR "renal function" OR "kidney elimination" OR "Renal Elimination" OR "creatinine clearance" OR "CCR" OR "CrCl" OR "eCCR" OR "inulin clearance" OR "cystatin C clearance" OR "urea clearance" OR "glomerulus filtration rate") |
| Scopus | Title / abstract / keyword | ("ventricular assist device*" OR "cardiac assist device*" OR "Heart-Assist Devices" OR "CF-LVAD" OR "continuous-flow pump*" OR f"pulsatile pump*" OR "heart assisting device*" OR "LVAD*" OR "CF-LVAD'" OR "cardiac assist device*" OR "ventricular assist device*" OR "ventricular-assist device*" ) AND ( "Glomerular Filtration Rate" OR "Kidney Function Test" OR "glomerular filtration" OR "GFR" OR "eGFR" OR "kidney function" OR "renal function" OR "kidney elimination" OR "Renal Elimination" OR "creatinine clearance" OR "CCR" OR "CrCl" OR "eCCR" OR "inulin clearance" OR "cystatin C clearance" OR "urea clearance" OR "glomerulus filtration rate") |
| Cochrane | All fields | ("heart-assist device" OR "heart assist device" OR "left ventricular assist device" OR "continuous-flow pump" OR "pulsatile pump" OR "heart assisting device" OR "LVAD" OR "CF-LVAD'" OR "cardiac assist device" OR "ventricular assist device" OR "ventricular-assist device") in Title Abstract Keyword AND ("Glomerular Filtration Rate" OR "Kidney Function Test" OR "glomerular filtration" OR "GFR" OR "eGFR" OR "kidney function" OR "renal function" OR "kidney elimination" OR "Renal Elimination" OR "creatinine clearance" OR "CCR" OR "CrCl" OR "eCCR" OR "inulin clearance" OR "cystatin C clearance" OR "urea clearance" OR "glomerulus filtration rate") |
| clinicaltrials.gov | With results | Condition: Keyword: "ventricular assist device*", "cardiac assist device*", "Heart-Assist Devices", "CF-LVAD", "continuous-flow pump*", "pulsatile pump*", LVAD, "left ventricular assist device*", "Glomerular Filtration Rate", "Kidney Function Tests", "Renal Elimination", "glomerular filtration", "GFR", "eGFR", "kidney function", "renal function", "kidney elimination", "creatinine clearance", "CCR", "CrCl", "eCCR", "inulin clearance", "cystatin C clearance", "urea clearance", and "glomerulus filtration rate"  Intervention: (heart-assist device) OR (heart assist device) OR (left ventricular assist device) OR (continuous-flow pump) OR (pulsatile pump) OR (heart assisting device) OR (LVAD) OR (CF-LVAD) OR (cardiac assist device) OR (ventricular assist device) |
| Manual search in Google and Google Scholar |  | Various permutations of keywords: "ventricular assist device*", "cardiac assist device*", "Heart-Assist Devices", "CF-LVAD", "continuous-flow pump*", "pulsatile pump*", LVAD, "left ventricular assist device*", "Glomerular Filtration Rate", "Kidney Function Tests", "Renal Elimination", "glomerular filtration", "GFR", "eGFR", "kidney function", "renal function", "kidney elimination", "creatinine clearance", "CCR", "CrCl", "eCCR", "inulin clearance", "cystatin C clearance", "urea clearance", and "glomerulus filtration rate" |

# Table S2. Characteristics of the studies included in meta-analysis.

| **Author, year** | **Study design** | **Country** | **No. of patients** | **Female (%)** | **Mean Age** | **Device type (%)** | **LVAD indication (%)** | **Etiology (%)** | **Mean LVEF** | **Baseline Cr** | **Post-LVAD renal replacement therapy (%)** | **eGFR method** | **Time period** | **Follow-up time points** |
| --- | --- | --- | --- | --- | --- | --- | --- | --- | --- | --- | --- | --- | --- | --- |
| Livingston et al [1], 2025 | Retrospective cohort | U.S. | 232 | 17% | 55.29 ± 14.34 | HeartMate II = 105 (45.3%)  HeartMate III = 59 (25.4%)  HVAD = 68 (29.3%) | NA | Ischemic = 85 (37%) | 15.98 ± 5.78 | 1.4 ± 0.61 | NA | NA | 2011-2020 | 1) 1 month  2) 3 months |
| Baker et al [2], 2025 | Retrospective cohort | U.S. | 160 | 18.1% | 57 (19-74) | HeartMate II = 89 (56%)  HeartWare = 61 (38%)  HeartMate III = 5 (3%)  Thoratec = 3 (2%)  HeartMate XVE = 1 (0.6%)  CentriMag = 1 (0.6%) | NA | Ischemic = 75 (47%)  non-ischemic = 85 (53%) | 17 | 1.6 | 22 (14%) | CKD-EPI | 2009-2019 | 1) 3 months  2) 12 months |
| Hamed et al. [3], 2025 | Retrospective cohort | Germany | 41 | 14.3% | 58.1 (50.8–64.4) | HeartMate III = 40 (98%)  HVAD = 1 (2%) | BTT = 30 (73%)  DT = 7 (17%)  BTD = 4 (10%) | Ischemic = 11 (27%)  non-ischemic = 30 (73%) | 15 (12–20) | 1.2 (0.95–1.47) | 5 (12.2%) | CKD-EPI | 2019-2023 | 1) 6 months  2) 12 months |
| Li et al. [4], 2025 | Prospective cohort | U.S. | 29 | 17.2% | 59.7 ± 11.2 | NA | BTT = 28 (96.6%)  DT = 1 (3.4%) | Ischemic = 16 (55.2%)  non-ischemic = 13 (44.8%) | 20.12 ± 4.08 | NA | 13 (44.8%) | CKD-EPI | NA | 1) 1 week  2) 2 weeks  3) 1 month  4) 3 months |
| Beargie et al. [5], 2023 | Prospective cohort | U.S. | 19 | 26.3% | 52.60 ± 12.80 | HeartWare (HVAD) = 15.3% HeartMate III= 78.9% | BTT = 15.8% BTD = 42.1% DT = 42.1% | Ischemic = 47.4% non-ischemic = 52.6% | 17.3 ± 6.6 | NA | NA | MDRD | 2020-2021 | 50.5 ± 30.9 days |
| Kakuda et al. [6], 2023 | Retrospective cohort | Japan | 132 | 28.8% | 41.85 ± 12.14 | HeartMate II = 31.8% EVAHEART = 18.1% Jarvik2000 = 14.4% DuraHeart = 12.1% Nipro-VAD = 4.5% HeartWare (HVAD) = 10.7% | BTT = 100% | NA | NA | NA | NA | NA | 2007-2022 | 3 years |
| Gündüz et al. [7], 2023 | Retrospective cohort | Turkey | 35 | 11.4% | 55.10 ± 9.09 | HeartMate II = 20.0% HeartMate III = 17.1% HeartWare (HVAD) = 62.9% | NA | NA | NA | NA | 100% | NA | 2012-2021 | 1 week 1 month 1 year |
| Jeon et al. [8], 2023 | Retrospective cohort | South Korea | 50 | 24.0% | 64.56 ± 13.14 | HeartMate II = 34.0% HeartWare (HVAD) = 66.0% | BTT = 56.0% DT = 44.0% | Ischemic = 26.0% non-ischemic = 74.0% -Dilated cardiomyopathy = 62.0% -Valvular heart disease = 6.0% -Hypertrophic cardiomyopathy = 4.0% -congenital heart disease = 2.0% | 23.17 ± 6.00 | 1.52 (1.12, 2.31) | 10% | CKD-EPI | 2012-2019 | 3 months 1 year 2 years 3 years |
| Grandin et al. [9], 2022 | Prospective cohort | Multinational (from STS database) | 9627 | 21.4% | 57.00 ± 12.88 | CF-LVAD = 25.1% | BTT = 41.8% | Ischemic = 48.7% non-ischemic = 51.3% | NA | NA | NA | NA | 2012-2017 | 1 month |
| Takada et al. [10], 2022 | Retrospective cohort | Turkey | 15 | 20.0% | NA | CF-LVAD = 100% | BTT = 100% | Ischemic = 6.7% non-ischemic = 93.3% -dilated cardiomyopathy = 73.3% -dilated phase hypertrophic cardiomyopathy = 13.3% -post myocarditis = 6.7% | 20.33 ± 6.56 | NA | NA | NA | 2011-2013 | 1 month |
| Wettersten et al. [11], 2021 | Prospective cohort | U.S. | 131 | 16.8% | 60.00 ± 13.00 | HeartWare (HVAD) = 26.7% HeartMate II = 61.8% HeartMate III = 11.5% | BTT = 42.8% | Ischemic = 64 (48.9%) non-ischemic = 67 (51.1%) | 19 ± 6 | 1.47 ± 0.48 | 0 (0%) | CKD-EPI | 2010-2017 | 1 month 3 months 6 months 1 year |
| Knudsen et al. [12], 2021 | Retrospective cohort | Denmark | 13 | 7.7% | 58.20 ± 10.30 | Heartmate II = 38.5%  HeartMate III = 61.5 % | BTD = 23.1 %  BTT = 46.2 %  DT = 30.8 % | Ischemic = 23.1%  Non-ischemic = 76.9 % | NA | 1.71 ± 0.74 | NA | CKD-EPI | 2006–2019 | 327 days [IQR  172–368] |
| Smail et al. [13], 2021 | Retrospective cohort | UK | 255 | 21.2% | 45.36 ± 13.42 | HeartMate II = 28.2% HeartWare (HVAD) = 71.0% | BTT = 100% | Ischemic = 19.2% non-ischemic = 76.9% -dilated cardiomyopathy = 73.7% -hypertrophic cardiomyopathy = 3.1% | NA | 1.26 ± 0.53 | NA | MDRD | 2006-2017 | 1 months  3 months 6 months 1 year  2 year 3 year |
| Gustafsson et al. [14], 2020 | Retrospective cohort | Kazakhstan and Europe | 194 | 13.9% | 54.00 ± 12.00 | HeartMate III = 100% | NA | Ischemic = 90 (46.4%) non-ischemic = 104 (53.6%) | 19 ± 6 | NA | NA | NA | beginning January 2015 in Kazakhstan beginning Europe in October 2015 | 6 months |
| Pinsino et al. [15], 2020 | Prospective cohort | U.S. | 83 | 14.5% | 60.10 ± 12.80 | Heartmate II = 26.5% Heartmate III = 71.1% HeartWare (HVAD) = 1.2% | DT = 72.3 % | Ischemic = 53.0% non-ischemic = 47.0% | NA | 1.27 [1.00, 1.60] | 1.2% | MDRD | 2016-2018 | 18 [16-26] days 151 [104-192] days 316 [288-348] days 671 [477-1377] days |
| Gürcü et al. [16], 2019 | Retrospective cohort | Turkey | 61 | 13.1% | 46.40 ± 11.20 | Heartmate II = 19.7% HeartWare (HVAD) = 37.7% Heartmate III = 42.6% | NA | Ischemic = 54.1% non-ischemic = 45.9% | 18.7 ± 3.4 | 0.9 ± 0.3 | NA | MDRD | 2015-2017 | 1 month 3 month 6 month |
| Quader et al. [17], 2019 | Prospective cohort | U.S. | 47 | 27.7% | 57.00 ± 12.40 | CF-LVAD = 100% | BTT: 57.4% DT = 36.2% | NA | 15.53 ± 6 | 1.84 ± 0.58 | 8.5% | MDRD | 2008-2013 | 1 month 3 month 6 month |
| Schmack et al. [18], 2018 | Retrospective cohort | Germany | 68 | 17.6% | 56.40 ± 9.43 | HeartWare (HVAD) = 100% | BTT = 67.6%) DT = 32.4% | Ischemic = 32.4% non-ischemic = 67.6% -dilated cardiomyopathy = 58.8% -hypertrophic non-obstructive cardiomyopathy = 2.9% -non-compaction cardiomyopathy = 1.5% |  | 1.56 ± 0.68 | NA | MDRD | 2010 - 2017 | 1 month |
| Nestorovic et al. [19], 2018 | Prospective cohort | Serbia | 47 | 6.4% | 51.90 ± 13.70 | HeartMate II = 74.5% HeartWare (HVAD) = 25.5% | BTT = 89.4% | Ischemic = 53.2% non-ischemic = 46.8% -idiopathic cardiomyopathy = 26% | 16.1 ± 4.9 | 1.12 ± 0.1 | NA | NA | 2013-2016 | 3 months 6 month 1 year |
| Ricklefs et al. [20], 2018 | Retrospective cohort | Germany | 103 | 23 (21.1%) | 53.80 ± 11.70 | NA | NA | NA | NA | 2.689 ± 2.01 | NA | MDRD | 2001–2016 | 62 ± 56 days |
| Verma et al. [21], 2017 | Retrospective cohort | U.S | 169 | 23.7% | 57.80 ± 14.00 | NA | BTT = 27.8% | NA | NA | NA | 3.6% | MDRD | 2010-2013 | 3 months 6 month 12 month 24 month |
| Yoshioka et al. [22], 2017 | Retrospective cohort | U.S. | 59 | 18.6% | 63.00 ± 13.00 | HeartMate II = 93.2% HeartWare (HVAD) = 6.8% | BTT = 32.2% DT = 67.8% | Ischemic cardiomyopathy = 53%  non-ischemic cardiomyopathy = 37% | 13.5 ± 4.6 | 1.51 ± 0.55 | 1.7% | MDRD | 2004-2015 | 1 month 6 month 1 year 2 year 3 year |
| Raichlin et al. [23], 2016 | Retrospective cohort | U.S. | 165 | 19.4% | 55.60 ± 13.50 | NA | BTT = 49.7% DT = 50.3% | Ischemic = 51.0% non-ischemic = 49.1% | 18.72 ± 11.2 | 1.3 ± 0.5 | 9.1% | MDRD | 2009-2014 | 1 month 3 month 6 month 1 year |
| LaRue et al. [24], 2015 | Retrospective study | U.S. | 50 | 20.0% | NA | Heartmate II = 94.0% HeartWare (HVAD) = 4.0% VentrAssist= 2.0% | BTT= 70.0% | Ischemic = 50.0% non-ischemic = 50.0% | NA | 1.09 ± 0.4 | NA | NA | 2008-2011 | 3-6 months |
| Grosman-Rimon et al. [25], 2015 | Prospective cohort | Canada | 9 | NA | 52.3 | HeartMate II = 66.7% HeartWare (HVAD) = 33.3% | BTT = 77.7% | Ischemic = 55.5% non-ischemic = 44.4% | 20.0 ± 1.6 | 1.37 ± 0.21 | NA | NA | NA | 3 month  6 month |
| Choudhary et al. [26], 2014 | Retrospective cohort | U.S. | 50 | 16.0% | 56.00 ± 10.90 | HeartMate II = 96.0% VentrAssist = 2.0% | NA | Ischemic = 62.0% non-ischemic = 38.0% -valvular = 2.0% -arrhythmic = 2.0% -congenital = 2.0% -hypertrophic cardiomyopathy = 2.0% | NA | 1.29 ± 0.43 | NA | NA | 2006-2012 | 6 months |
| Demirozu et al. [27], 2014 | Retrospective cohort | U.S. | 23 | 17.4% | 47.16 ± 16.00 | HeartMate II = 100% | BTT = 73.9% DT = 20.1% | Ischemic cardiomyopathy = 30.44%  Idiopathic cardiomyopathy = 69.56% | 19 ± 5 | 1.40 ± 0.6 | 17.4% | CrCl | 2003 - 2012 | 3 months 6 month 1 year |
| Brisco et al. [28], 2014 | Retrospective cohort | U.S. | 3363 | 21.7% | 54.50 ± 13.80 | LVAD alone = 92.1% CF-LVAD = 79.3% | DT = 17.7% | Ischemic = 45% non-ischemic = 55% | NA | 1.49 ± 0.83 | NA | MDRD | 2006 - 2011 | 1 month |
| Jacobs et al. [29], 2014 | Retrospective cohort | Belgium | 61 | 14.8% | 51.63 ± 14.68 | Synergy micropump = 29.5% | NA | Ischemic = 50.8% non-ischemic = 49.2% -dilated cardiomyopathy = 49.2% | NA | 1.39 ± 0.48 | NA | MDRD | 2007-2013 | 3 months |
| Hasin et al. [30], 2012 | Retrospective cohort | U.S. | 83 | 18.1% | 63.00 ± 12.30 | Heartmate II = 100% | BTT = 32.5% DT = 67.5% | Ischemic = 55.4% non-ischemic = 44.6% | 19.8 ± 8.6 | 1.6 ± 0.7 | 9.7% | MDRD | 2007-2010 | 1 month 3 months 6 months |
| Iwashima et al. [31], 2012 | Prospective cohort | Japan | 110 | 27.3% | 34.80 ± 13.10 | Nipro = 93.7% Novacor = 3.6% Heart Mate I = 2.7% | NA | Ischemic = 7.3% non-ischemic = 92.7% | NA | 1.41 ± 1.00 | NA | Cockcroft-Gault formula | 1994-2009 | 2 weeks |
| Sandner et al. [32], 2009 | Retrospective cohort | Austria | 86 | 15.1% | 52.60 ± 11.60 | DeBakey VAD = 87.2% HeartWare (HVAD) = 7.0% DuraHeart LVAD = 5.8% | BTT = 52.33% | Ischemic = 31.4% non-ischemic = 68.6% | NA | 1.28 ± 0.41 | 34.9% | MDRD | NA | 1 month |
| Kamdar et al. [33], 2009 | Retrospective cohort | U.S. | 58 | 24.1% | 51.89 ± 12.90 | VentrAssist = 17.2% HeartMate II = 51.2% HeartMate XVE (Pulsatile flow) = 31.0% | BTT = 58 (100%) | Ischemic = 58.6% non-ischemic = 41.4% | 15.88 ± 5.31 | 1.78 ± 3.06 | NA | CrCl | NA | 1 month 3 months |
| Maeder et al. [34], 2009 | Prospective cohort | Australia | 20 | 20.0% | 57.00 ± 17.00 | VentrAssist = 100% | NA | Ischemic = 50.0% non-ischemic = 50.0% | 18 ± 8 | 1.89 ± 0.95 | NA | MDRD | NA | 140 days  [34–367] |
| Ma et al. [35], 2008 | Retrospective cohort | Japan | 28 | 17.9% | 38.00 ± 17.00 | pulsatile LVAD = 100% | NA | Ischemic = 14.3%  non-ischemic = 67.9% | NA | 1.34 ± 0.82 | 14.3% | Creatinine clearance | 2002-2007 |  |
| Sandner et al. [36], 2008 | Retrospective cohort | Austria | 92 | 31.5% | 58.60 ± 7.80 | Pulsatile LVAD = 100% | BTT = 51.7% | Ischemic = 37.9% non-ischemic = 62.0% | NA | 1.65 ± 0.6 | 35.9% | MDRD | NA | 2 weeks 1 month 3 month 196.4 ± 116.4 days |
| Radovancevic et al. [37], 2007 | Prospective cohort | U.S. | 70 | 10.0% | 50.51 ± 14.11 | CF-LVAD = 17.1% pulsatile-flow LVAD = 82.9% | NA | NA | 18 ± 5 | NA | NA | CrCl | NA | 6 months 1 year |
| Butler et al. [38], 2006 | Retrospective cohort | U.S. | 220 | 13.2% | 51.00 ± 12.00 | Novacor = 100% | BTT = 85.5% | Ischemic = 48.1% non-ischemic = 51.8% | NA | 1.5 ± 0.8 | NA | Cockcroft-Gault formula | NA | 2 weeks 4 weeks |

**Abbreviation:** LVAD=left ventricular assist device; Cr=creatinine; eGFR=estimated glomerular filtration rate; U.S.=United States; MDRD=The original Modification of Diet in Renal Disease; BTT: bridge to transplant; DT: destination therapy; BTD=bridge to decision; CrCl=creatinine clearance rate; CKD-EPI=The Chronic Kidney Disease Epidemiology Collaboration; CF-LVAD=Continuous-flow left ventricular assist device; NA=not available; HVAD=HeartWare Ventricular Assist Device; VAD=ventricular assist device; LVEF=Left ventricular ejection fraction.

# Table S3. Risk of bias assessment using the NIH Quality Assessment Tool for Before-After (Pre-Post) Studies with No Control Group.

| **Study** | **Q1** | **Q2** | **Q3** | **Q4** | **Q5** | **Q6** | **Q7** | **Q8** | **Q9** | **Q10** | **Q11** | **Q12** | **Yes / Applicable** | **Quality** |
| --- | --- | --- | --- | --- | --- | --- | --- | --- | --- | --- | --- | --- | --- | --- |
| **Livingston 2025** | Y | Y | Y | N | Y | Y | Y | NA | N | Y | N | NA | 7/10 | Fair |
| **Baker 2025** | Y | Y | Y | Y | Y | Y | Y | NA | Y | Y | N | NA | 9/10 | Good |
| **Hamed 2025** | Y | Y | N | N | Y | Y | Y | NA | Y | N | N | NA | 6/10 | Fair |
| **Li 2025** | Y | Y | N | CD | N | Y | Y | NA | Y | N | N | NA | 5/10 | Fair |
| **Beargie 2023** | Y | Y | CD | CD | N | Y | Y | NA | N | Y | N | NA | 5/10 | Fair |
| **Kakuda 2023** | Y | Y | N | Y | Y | Y | Y | NA | Y | Y | N | NA | 7/10 | Fair |
| **Gündüz 2023** | Y | Y | Y | N | CD | Y | Y | NA | N | N | N | NA | 5/10 | Fair |
| **Jeon 2023** | Y | Y | CD | CD | N | Y | Y | NA | CD | Y | N | NA | 5/10 | Fair |
| **Grandin 2022** | Y | Y | N | N | Y | Y | Y | NA | CD | N | N | NA | 5/10 | Fair |
| **Takada 2022** | Y | Y | N | N | N | Y | Y | NA | Y | N | N | NA | 5/10 | Fair |
| **Wettersten 2021** | Y | Y | Y | Y | Y | Y | Y | NA | N | Y | N | NA | 8/10 | Good |
| **Knudsen 2021** | Y | CD | N | N | N | Y | Y | NA | N | Y | N | NA | 4/10 | Poor |
| **Smail 2021** | Y | Y | Y | Y | Y | Y | Y | NA | N | Y | N | NA | 8/10 | Good |
| **Gustafsson 2020** | Y | Y | N | N | Y | Y | N | NA | Y | N | N | NA | 5/10 | Fair |
| **Pinsino 2020** | Y | Y | CD | N | Y | Y | Y | NA | N | Y | N | NA | 6/10 | Fair |
| **Gürcü 2019** | Y | Y | Y | Y | Y | Y | Y | NA | N | Y | N | NA | 8/10 | Good |
| **Quader 2020** | Y | Y | N | Y | N | Y | Y | NA | Y | Y | N | NA | 7/10 | Fair |
| **Schmack 2018** | Y | Y | Y | Y | Y | Y | Y | NA | Y | Y | N | NA | 9/10 | Good |
| **Nestorovic 2018** | Y | Y | Y | Y | N | Y | Y | NA | N | Y | N | NA | 7/10 | Fair |
| **Ricklefs 2018** | Y | Y | N | Y | Y | Y | Y | NA | N | Y | N | NA | 7/10 | Fair |
| **Verma 2017** | Y | Y | N | N | Y | Y | Y | NA | N | Y | N | NA | 6/10 | Fair |
| **Yoshioka 2017** | Y | Y | N | N | N | Y | Y | NA | Y | Y | N | NA | 6/10 | Fair |
| **Raichlin 2016** | Y | Y | Y | Y | Y | Y | Y | NA | N | Y | N | NA | 8/10 | Good |
| **LaRue 2015** | Y | Y | N | N | N | Y | Y | NA | Y | Y | N | NA | 6/10 | Fair |
| **Grosman-Rimon 2015** | Y | Y | N | N | N | Y | Y | NA | N | N | N | NA | 4/10 | Poor |
| **Choudhary 2014** | Y | Y | N | N | N | Y | Y | NA | N | Y | N | NA | 5/10 | Fair |
| **Demirozu 2014** | Y | Y | N | N | N | Y | Y | NA | Y | Y | N | NA | 6/10 | Fair |
| **Brisco 2014** | Y | Y | Y | N | Y | Y | Y | NA | N | Y | N | NA | 7/10 | Fair |
| **Jacobs 2014** | Y | Y | Y | N | N | Y | Y | NA | Y | Y | N | NA | 7/10 | Fair |
| **Hasin 2012** | Y | Y | Y | Y | Y | Y | Y | NA | N | Y | N | NA | 8/10 | Good |
| **Iwashima 2012** | Y | Y | N | N | Y | Y | Y | NA | Y | Y | N | NA | 7/10 | Fair |
| **Sandner 2009** | Y | Y | Y | Y | Y | Y | Y | NA | N | Y | N | NA | 8/10 | Good |
| **Kamdar 2009** | Y | Y | N | N | N | Y | Y | NA | Y | Y | N | NA | 6/10 | Fair |
| **Maeder 2009** | Y | Y | N | N | N | Y | Y | NA | N | Y | N | NA | 5/10 | Fair |
| **Ma 2008** | Y | Y | N | CD | N | N | Y | NA | Y | N | N | NA | 4/10 | Poor |
| **Sandner 2008** | Y | Y | Y | Y | Y | Y | Y | NA | N | Y | N | NA | 8/10 | Good |
| **Radovancevic 2007** | Y | Y | N | N | N | Y | Y | NA | Y | Y | N | NA | 6/10 | Fair |
| **Butler 2006** | Y | Y | Y | Y | Y | Y | Y | NA | N | Y | N | NA | 8/10 | Good |

**NIH item definitions**

- **Q1: Was the study question or objective clearly stated?**
- **Q2: Were eligibility/selection criteria prespecified and clearly described?**
- **Q3: Were participants representative of those who would be eligible for the intervention in the clinical population of interest?**
- **Q4: Were all eligible participants that met the prespecified entry criteria enrolled?**
- **Q5: Was the sample size sufficiently large to provide confidence in the findings?**
- **Q6: Was the intervention clearly described and delivered consistently across the study population?**
- **Q7: Were the outcome measures prespecified, clearly defined, valid, reliable, and assessed consistently across all participants?**
- **Q8: Were outcome assessors blinded to the intervention status of participants?**
- **Q9: Was the loss to follow-up after baseline 20% or less, or adequately accounted for in the analysis?**
- **Q10: Did the statistical methods examine changes in outcome measures from before to after the intervention?**
- **Q11: Were outcome measures taken multiple times before and after the intervention?**
- **Q12: If the intervention was conducted at the group level, did the statistical analysis account for clustering?**

**Scoring method for this review:**

- Q8 was considered not applicable because renal outcomes were objective laboratory-based measures.
- Q12 was considered not applicable because the intervention was not delivered at the cluster level.
- Therefore, scores were based on 10 applicable items.
- Each Yes = 1 point
- No / Cannot determine = 0 points

**Quality grading: Poor:** <50% / **Fair:** 50-75% **/ Good:** ≥75%

# Table S4. Sensitivity analysis restricted to studies with fair or good risk of bias.

| **Follow-up time** | **Pooled mean change in eGFR**  **β (95% CI)** | **p-value** |
| --- | --- | --- |
| 1 week | 19.15 (13.18 to 25.13) | <0.001 |
| 2 weeks | 18.48 (12.04 to 24.92) | <0.001 |
| 1 month | 23.97 (19.41 to 28.53) | <0.001 |
| 3 months | 15.31 (10.74 to 19.88) | <0.001 |
| 6 months | 8.23 (3.66 to 12.79) | <0.001 |
| 9 months | 8.40 (1.06 to 15.74) | 0.025 |
| 1 year | 7.54 (2.75 to 12.33) | 0.002 |
| 2 years | 3.72 (-2.07 to 9.51) | 0.208 |
| 3 years | 3.19 (-3.37 to 9.76) | 0.340 |

# Table S5. Multivariate meta-regression of mean age and female proportion in relation to change in eGFR across follow-up.

| **Follow-up time** | **Age per 10-year increase, β (95% CI)** | **p-value** | **Female per 10 percentage-point increase, β (95% CI)** | **p-value** |
| --- | --- | --- | --- | --- |
| 1 week | -3.61 (-13.26 to 6.04) | 0.463 | 2.75 (-6.49 to 12.00) | 0.560 |
| 2 weeks | -10.82 (-19.93 to -1.72) | 0.020 | 3.60 (-5.89 to 13.09) | 0.457 |
| 1 month | -7.58 (-15.37 to 0.21) | 0.057 | 5.43 (-3.04 to 13.89) | 0.209 |
| 3 months | 0.58 (-6.96 to 8.13) | 0.879 | 8.83 (0.91 to 16.75) | 0.029 |
| 6 months | 0.03 (-7.55 to 7.61) | 0.995 | 6.76 (-0.98 to 14.50) | 0.087 |
| 9 months | 0.85 (-13.42 to 15.11) | 0.908 | 7.73 (-3.63 to 19.09) | 0.182 |
| 1 year | -3.24 (-11.14 to 4.66) | 0.422 | 1.75 (-6.83 to 10.33) | 0.689 |
| 2 years | -0.84 (-9.36 to 7.68) | 0.846 | 7.46 (-7.10 to 22.01) | 0.315 |
| 3 years | -1.72 (-9.59 to 6.16) | 0.669 | 3.99 (-16.63 to 24.61) | 0.704 |


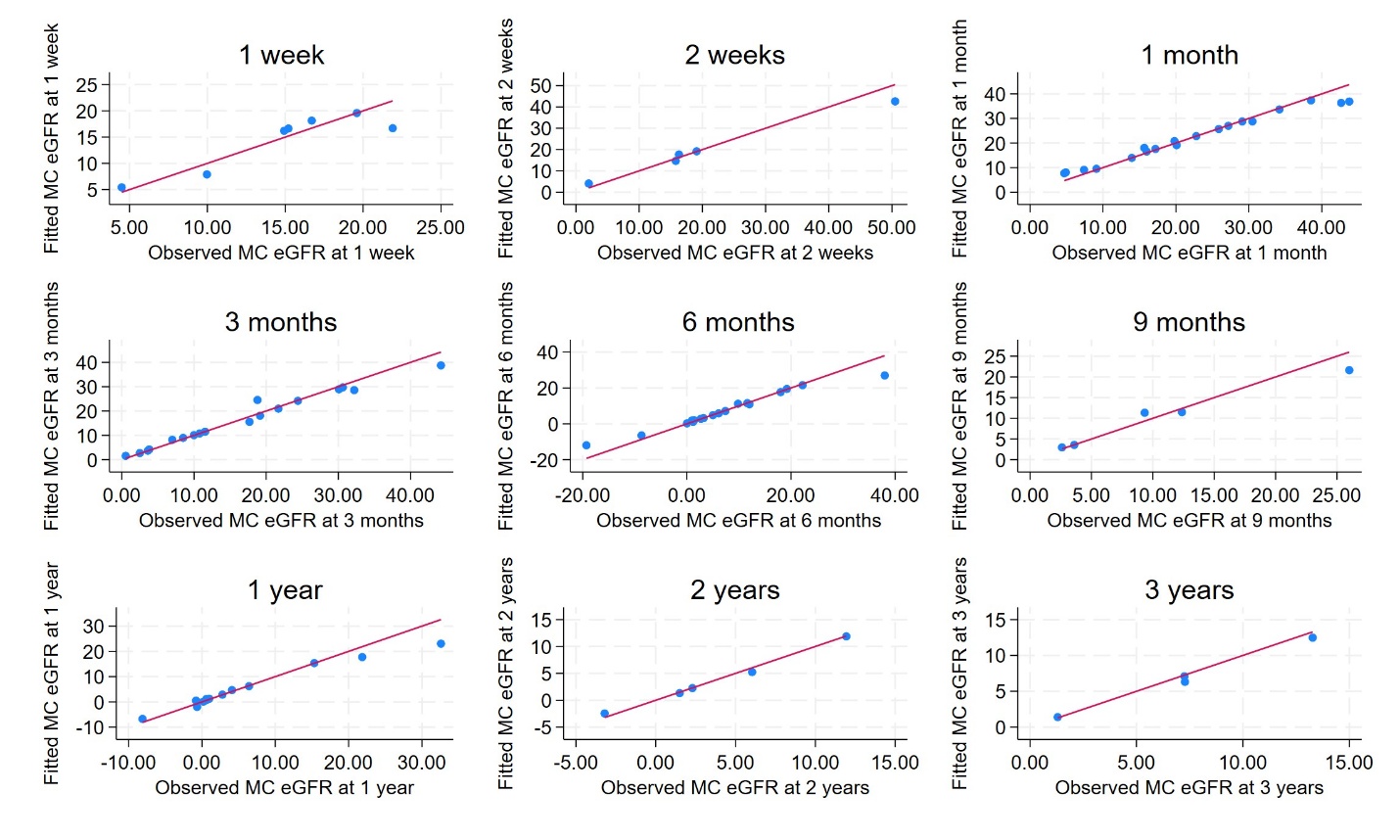


# Figure S1. Observed-versus-fitted plots for the multivariate random-effects meta-analysis.


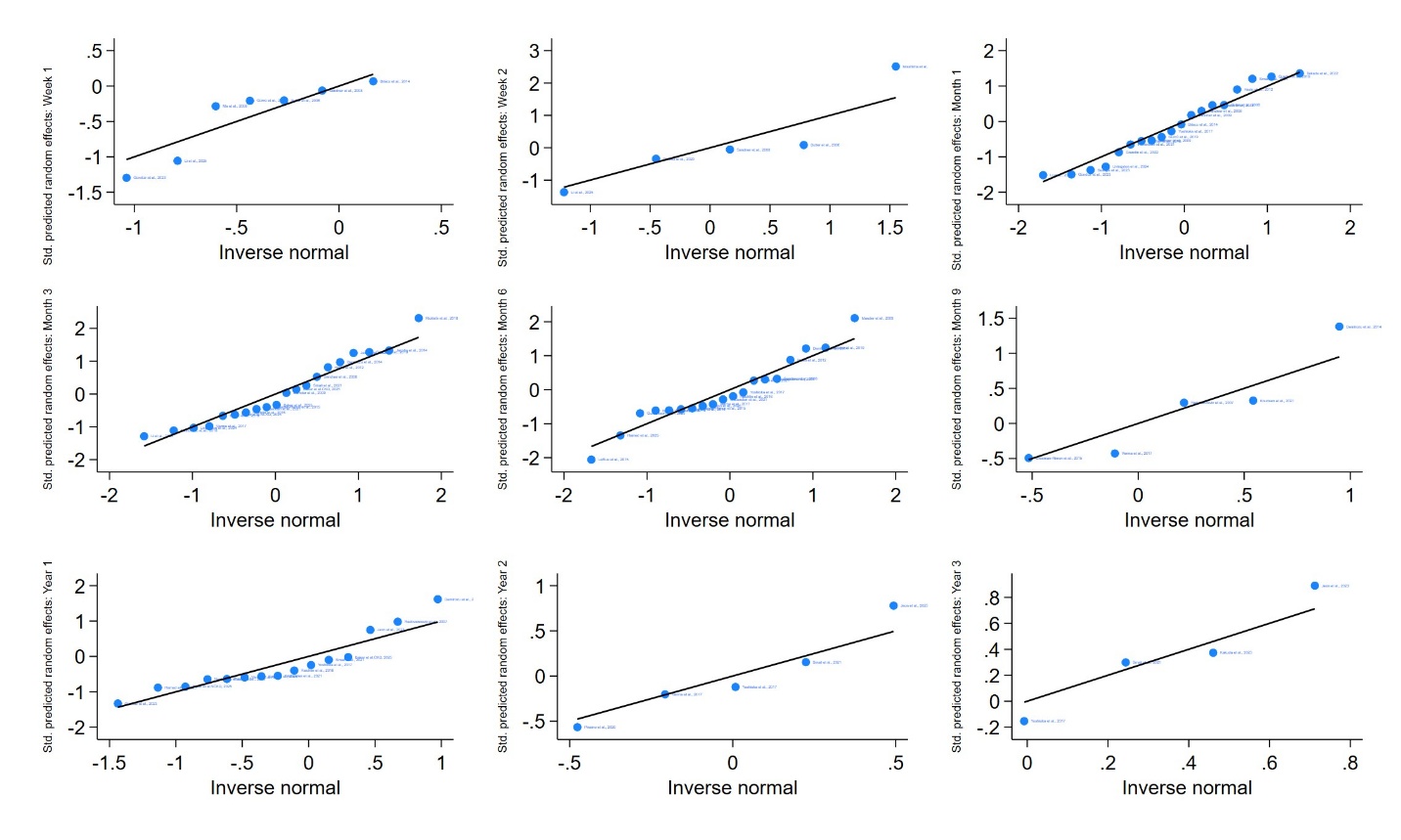


# Figure S2. Q-Q plots of standardized predicted random effects from the multivariate random-effects meta-analysis.


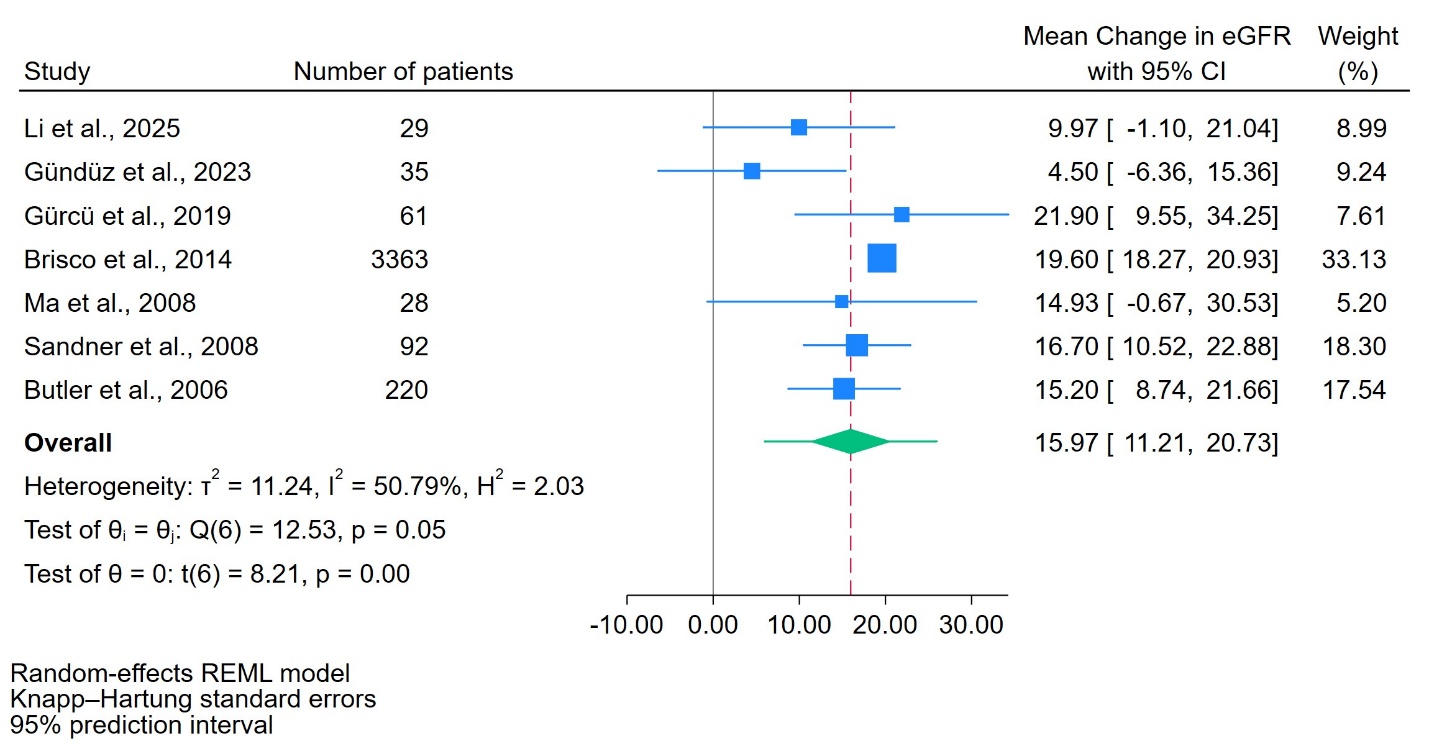


# Figure S3. Forest plot of the random-effects meta-analysis showing the pooled mean change in eGFR at 1 week.


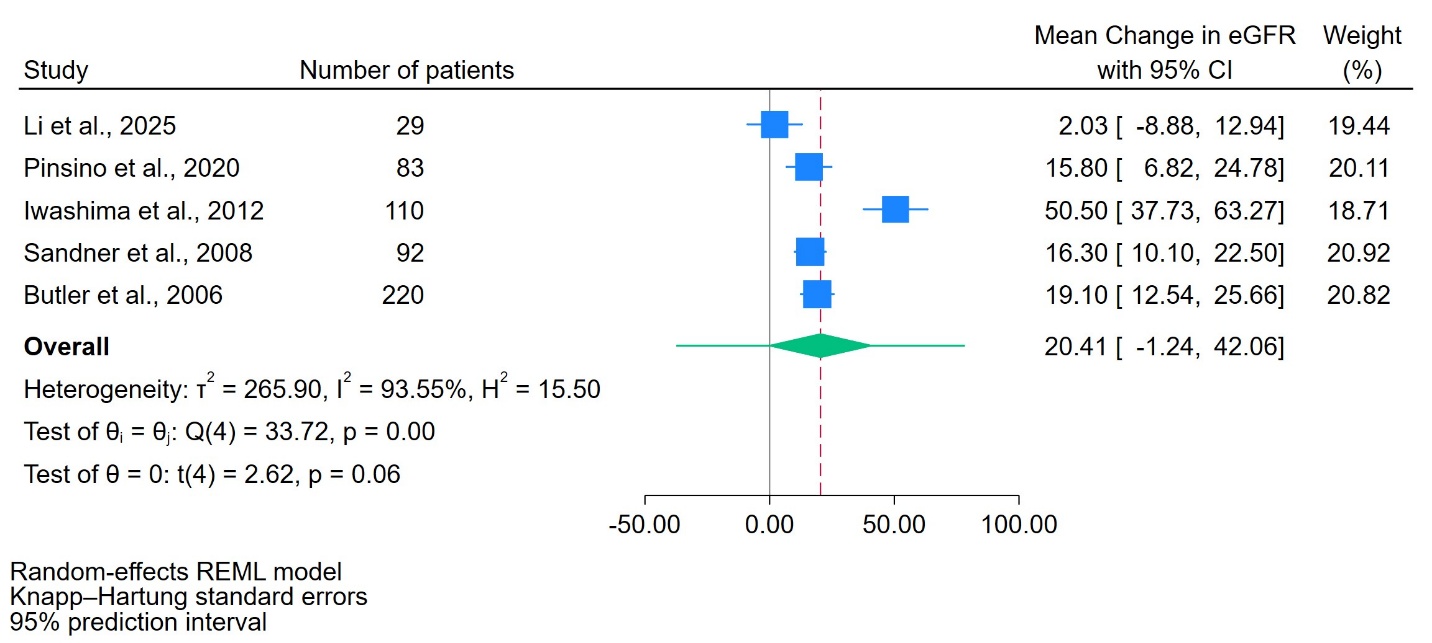


# Figure S4. Forest plot of the random-effects meta-analysis showing the pooled mean change in eGFR at 2 weeks.


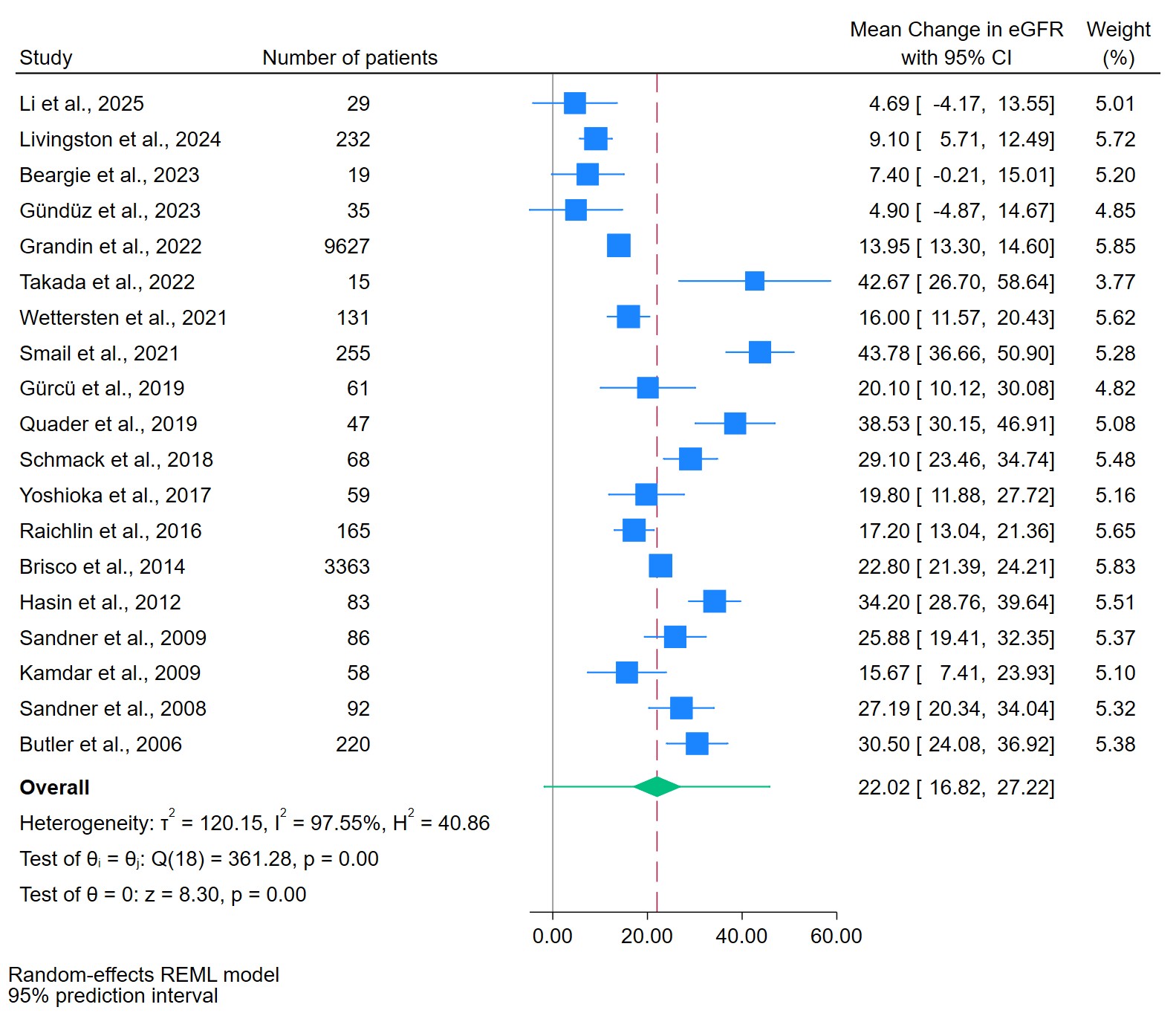


# Figure S5. Forest plot of the random-effects meta-analysis showing the pooled mean change in eGFR at 1 month.


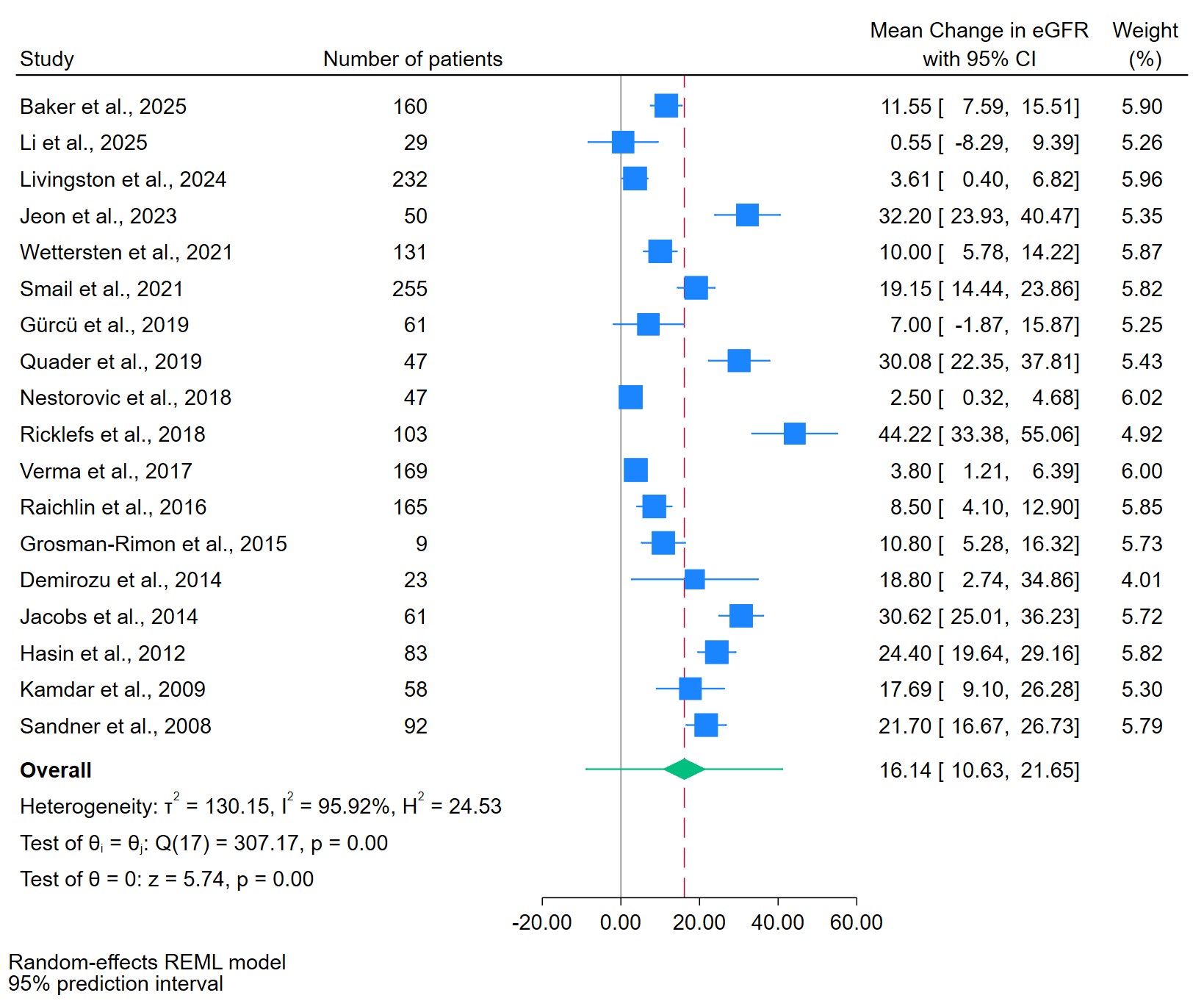


# Figure S6. Forest plot of the random-effects meta-analysis showing the pooled mean change in eGFR at 3 months.


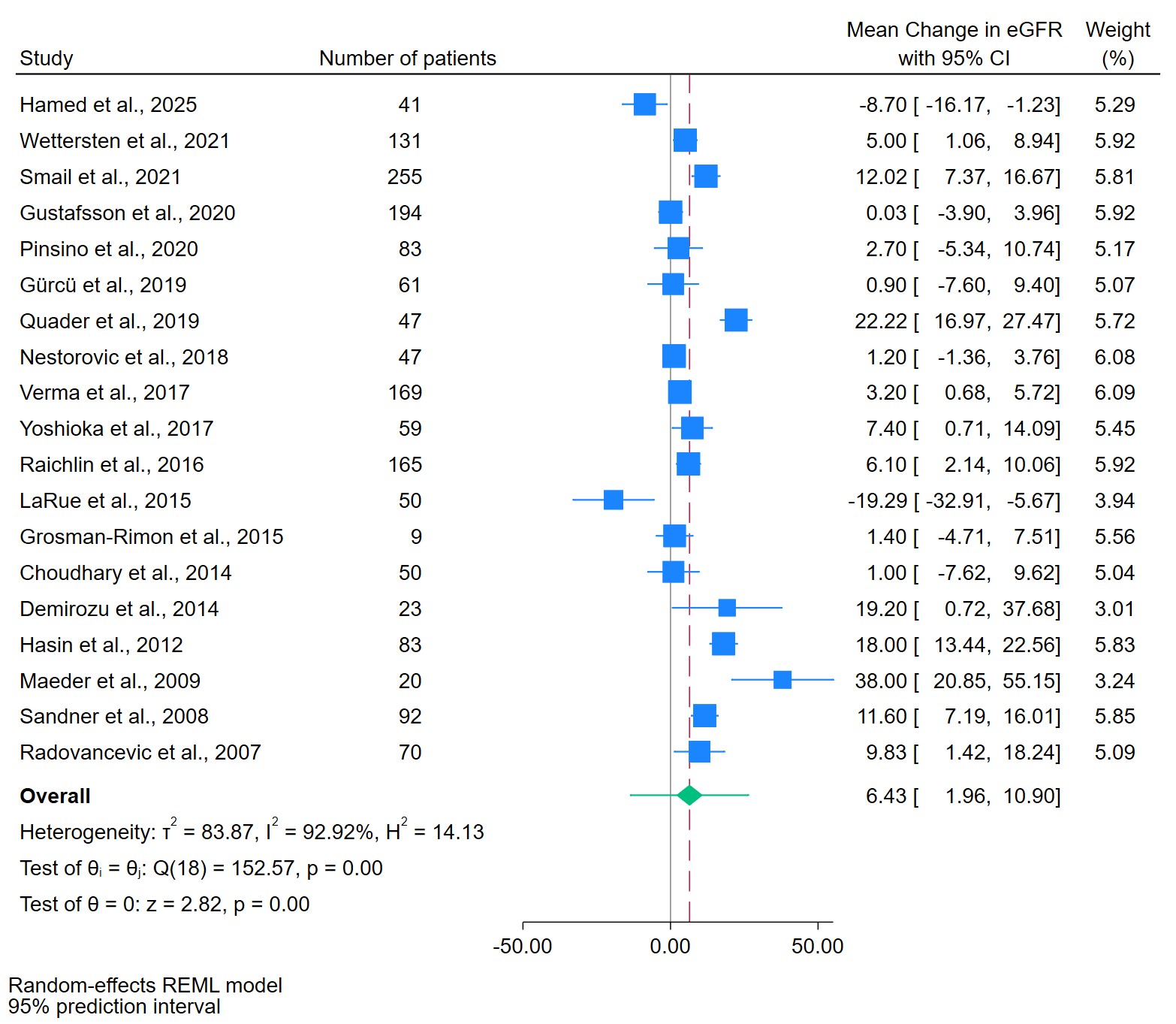


# Figure S7. Forest plot of the random-effects meta-analysis showing the pooled mean change in eGFR at 6 months


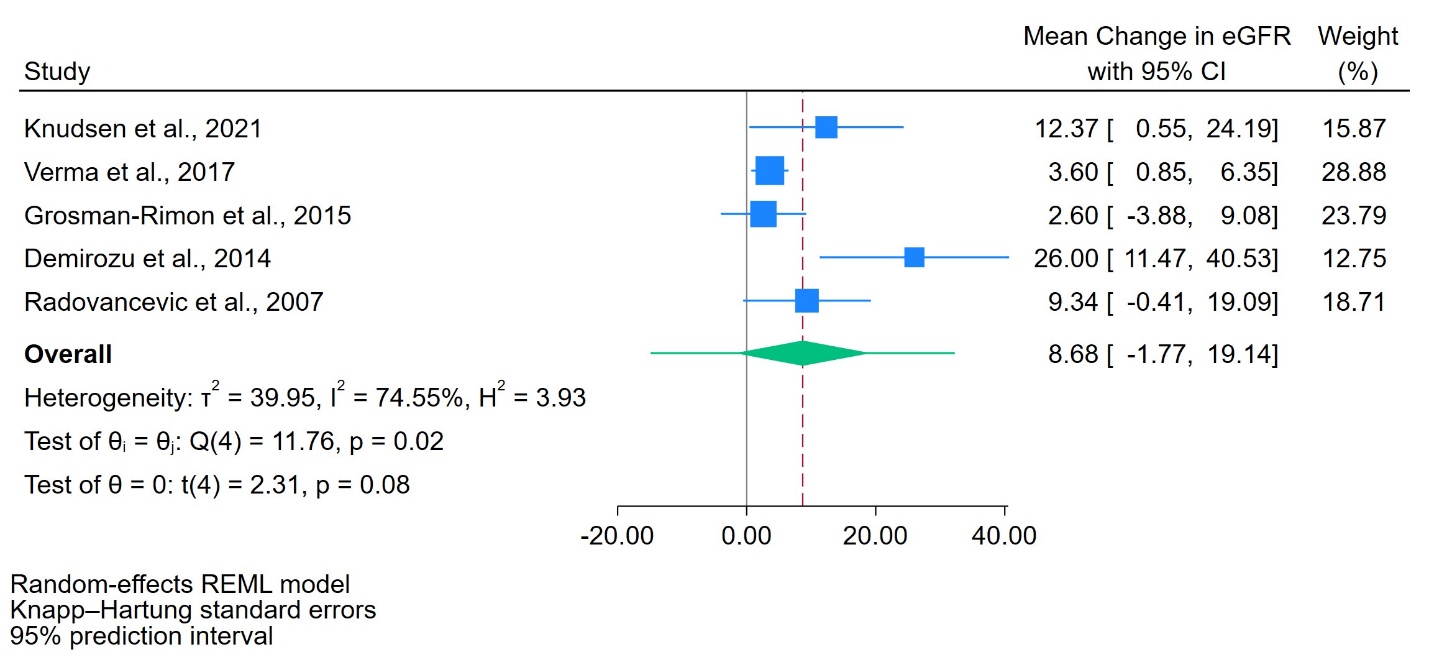


# Figure S8. Forest plot of the random-effects meta-analysis showing the pooled mean change in eGFR at 9 months.


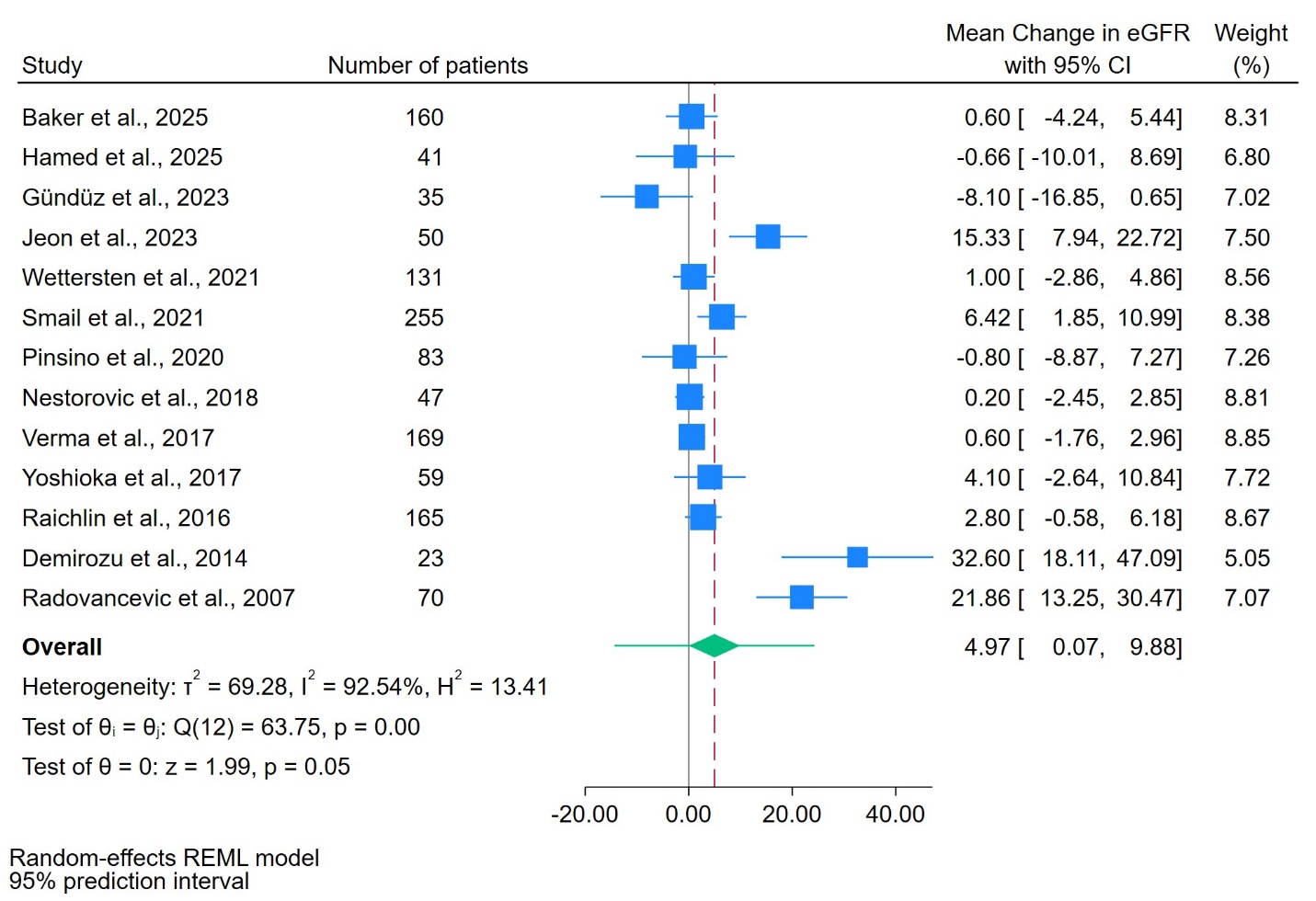


# Figure S9. Forest plot of the random-effects meta-analysis showing the pooled mean change in eGFR at 1 year.


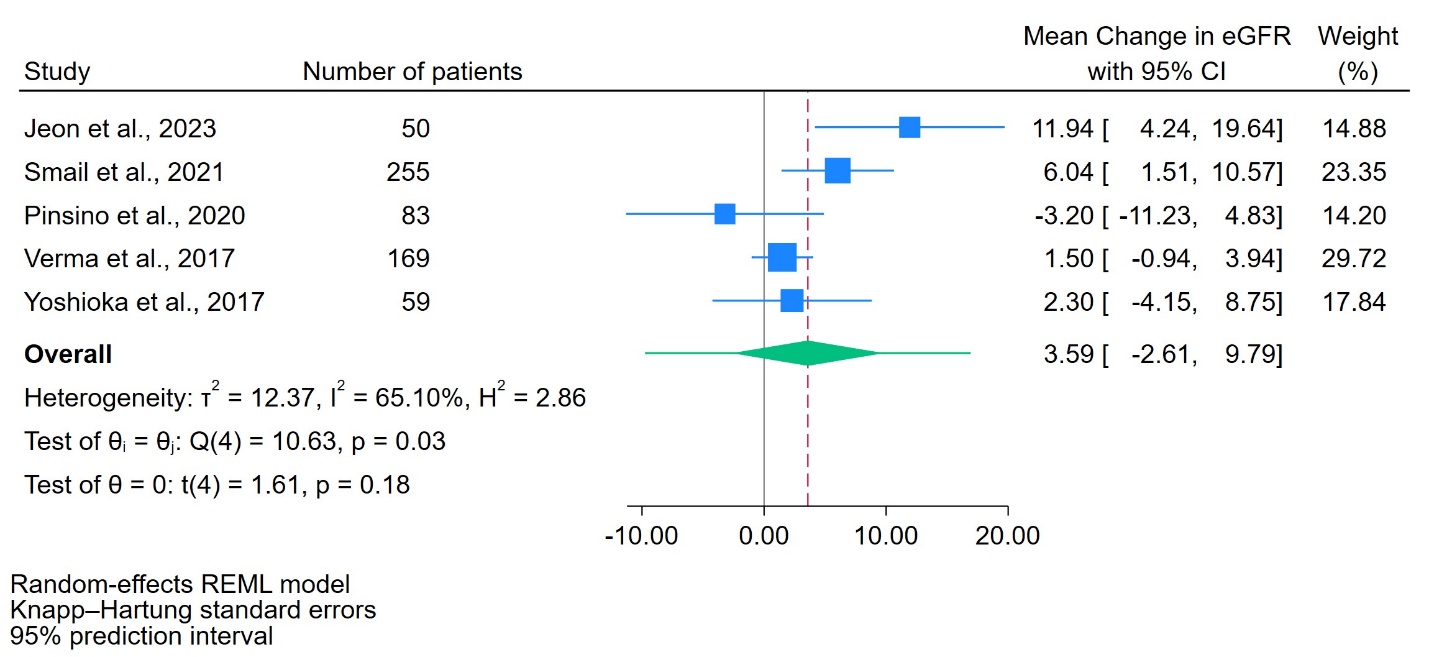


# Figure S10. Forest plot of the random-effects meta-analysis showing the pooled mean change in eGFR at 2 years.


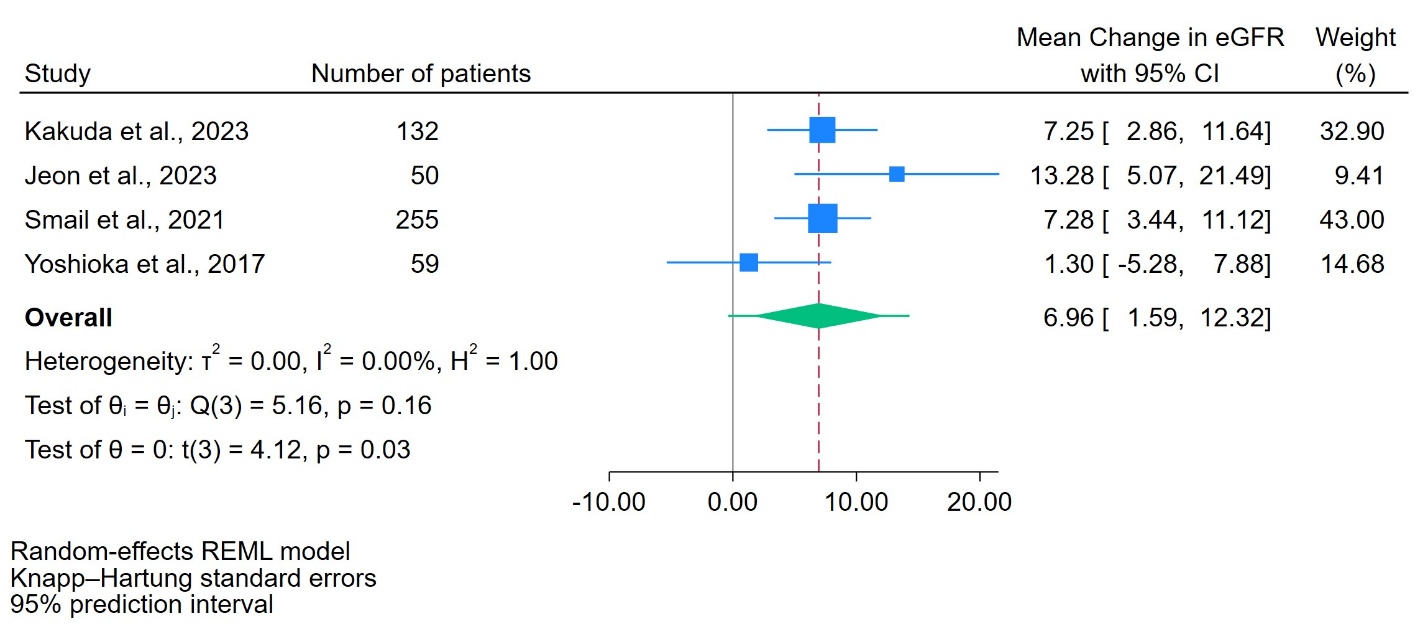


# Figure S11. Forest plot of the random-effects meta-analysis showing the pooled mean change in eGFR at 3 years.


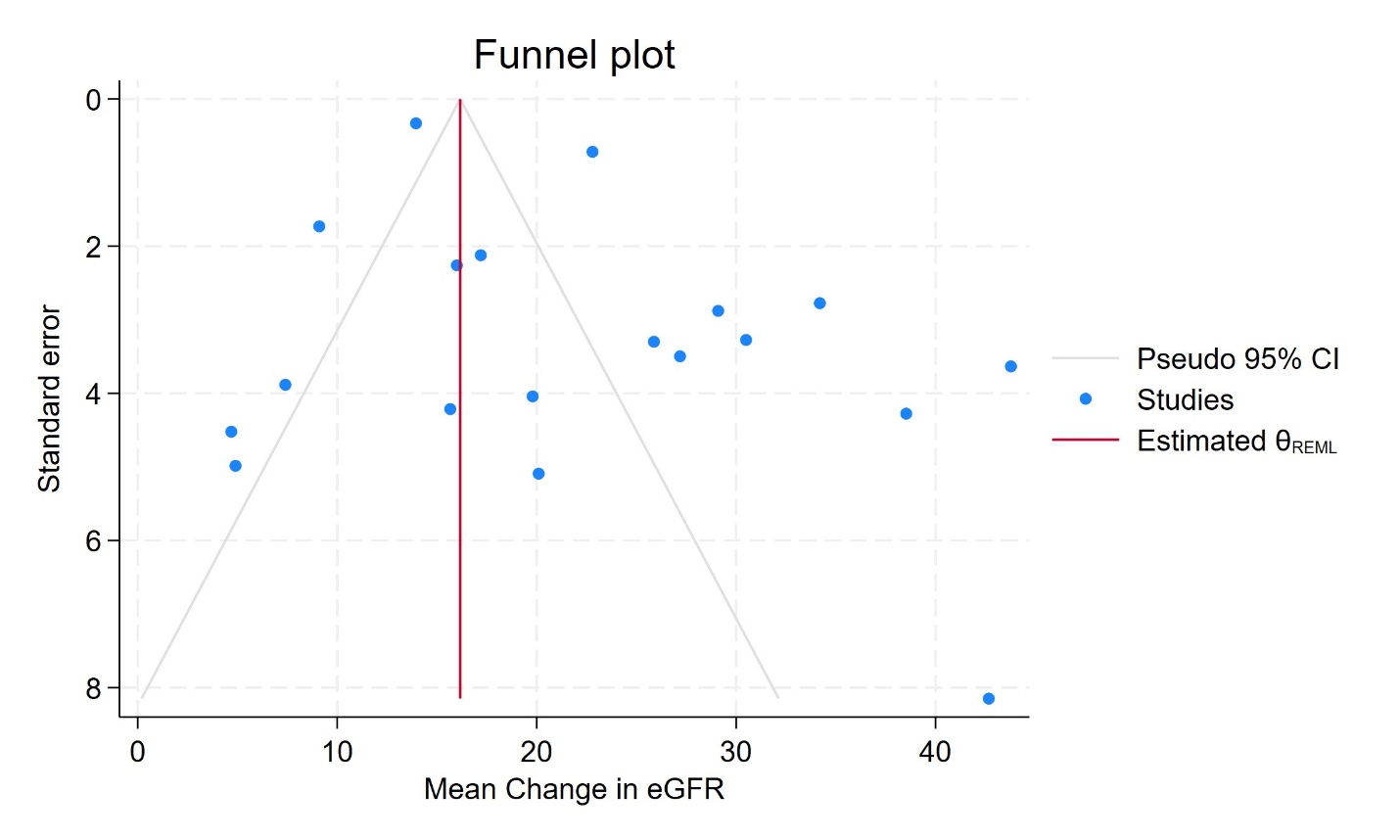


# Figure S12. Funnel plot for assessment of small-study effects in the random-effects meta-analysis of mean change in eGFR at 1 month.


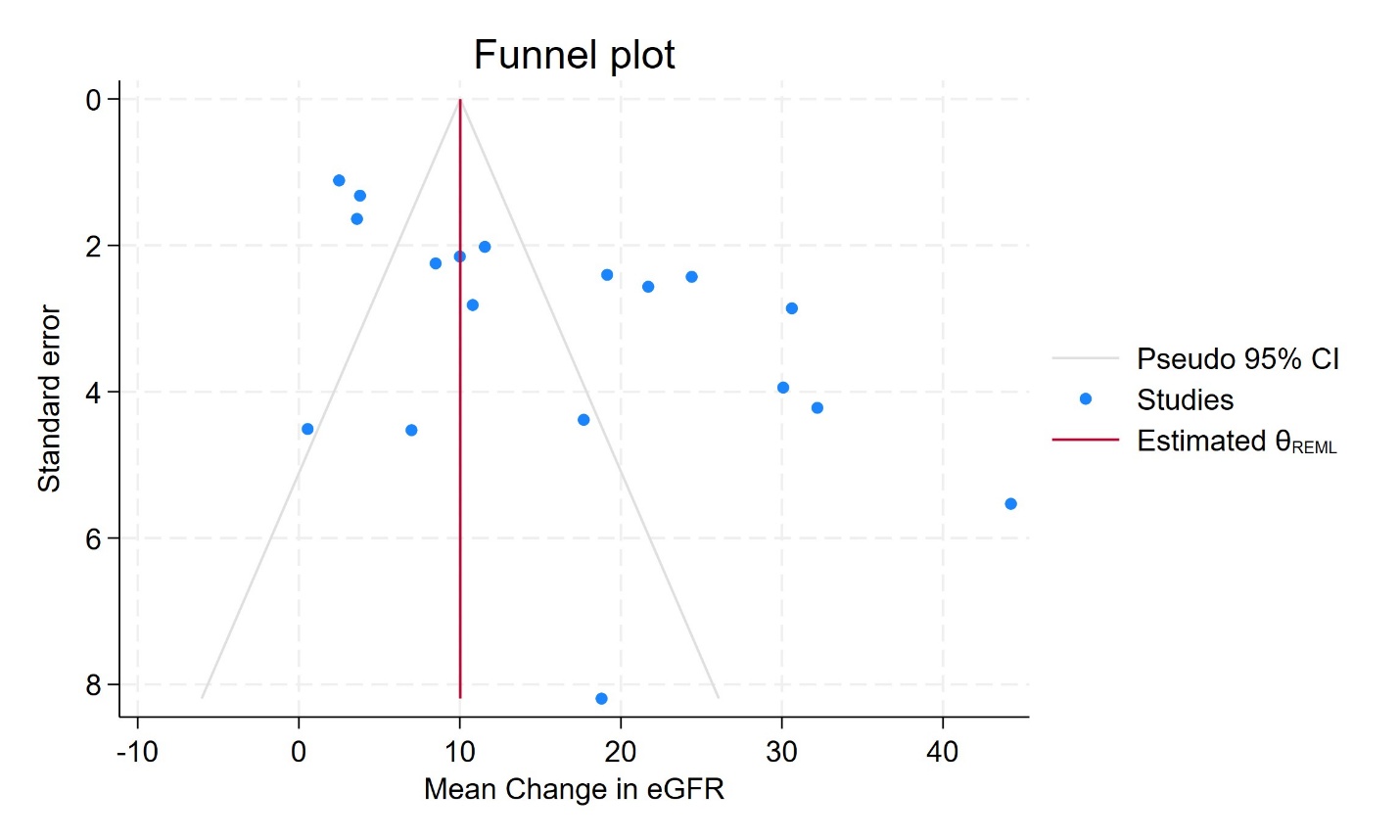


# Figure S13. Funnel plot for assessment of small-study effects in the random-effects meta-analysis of mean change in eGFR at 3 months.


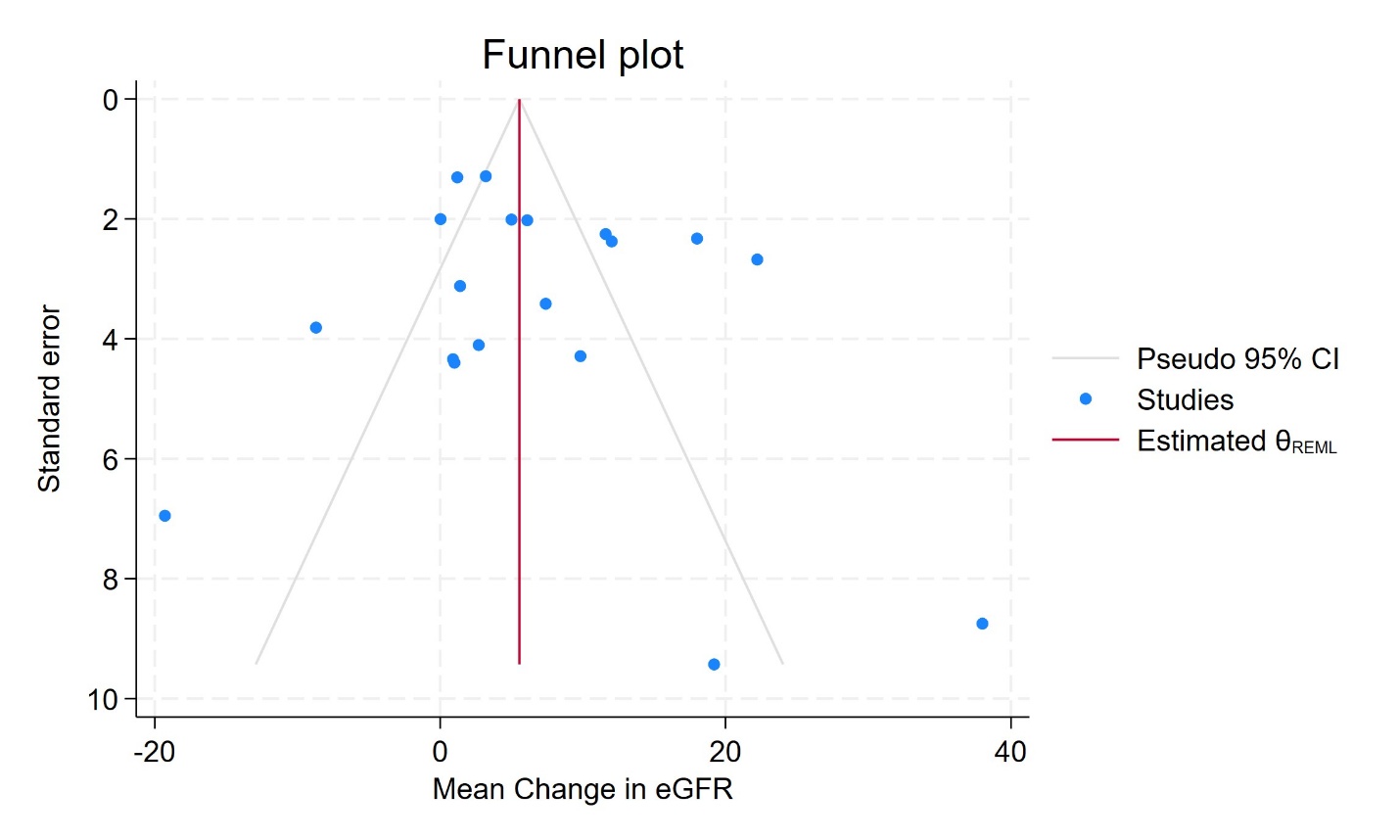


# Figure S14. Funnel plot for assessment of small-study effects in the random-effects meta-analysis of mean change in eGFR at 6 months.


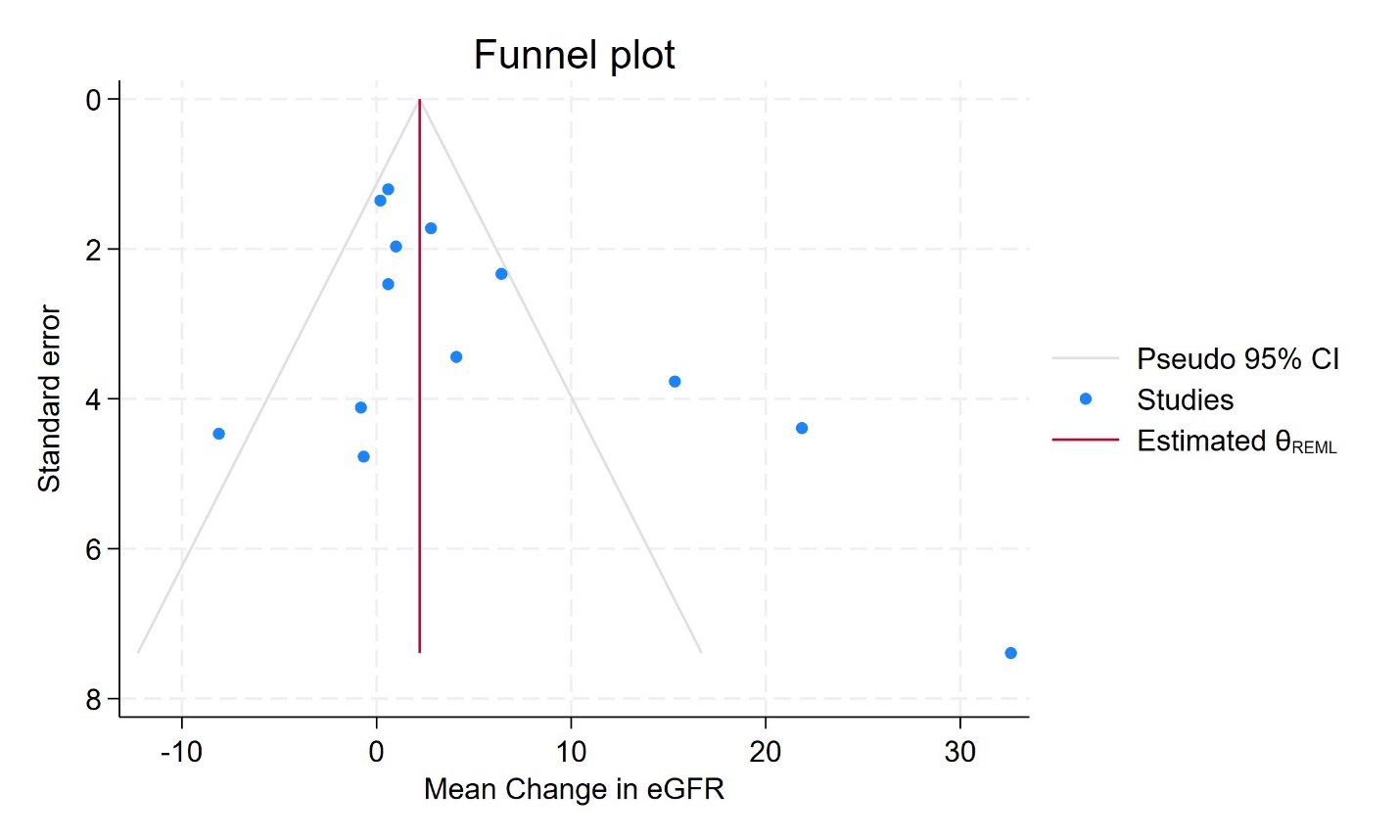


# Figure S15. Funnel plot for assessment of small-study effects in the random-effects meta-analysis of mean change in eGFR at 1 year.
